# The lagged and cumulative effects of permafrost and seasonally frozen soil on water use efficiency in the Qilian Mountains, Northwest China

**DOI:** 10.1016/j.isci.2026.117973

**Published:** 2026-10-29

**Authors:** Lili Li, Yu Guo, Yao Zhang, Duni Jia, Jia Du, Yong Gu, Tianxu Luo, Jinshan Li, Dawei Wang

**Affiliations:** 1 College of Surveying and Information Engineering, West Yunnan University of Applied Sciences, Dali 671006, People's Republic of China; 2 Northwest Institute of Eco-Environment and Resources, Chinese Academy of Sciences, Lanzhou 730000, People's Republic of China; 3 College of Geography and Environmental Science, Northwest Normal University, Lanzhou, Gansu 730070, People's Republic of China; 4 Wuwei Bureau of Forestry and Grassland Administration, Wuwei 733000, People's Republic of China

**Keywords:** lagged and cumulative effects, permafrost, seasonally frozen soil, self-calibrated Palmer drought severity index, scPDSI, Qilian Mountains

## Abstract

Permafrost in the Qilian Mountains is highly sensitive to climate change, affecting ecosystem energy, water, and carbon cycling. Using MODIS data and the self-calibrated Palmer Drought Severity Index (scPDSI), we analyzed WUE dynamics and drought responses in permafrost and seasonally frozen soil regions during 2001–2020. WUE showed an overall gradual decline and an intra-annual increase-decrease–increase pattern, with growth rates peaking in November and the lowest values occurring in June. A threshold of 0.6 gC m^−2^ mm^−1^ demarcated the two frozen-soil types. Delayed drought responses occurred in 50.77% of vegetation, with a mean lag of 6.82 months; seasonally frozen soil mainly showed non-lagged and long-term responses, whereas permafrost mainly showed non-lagged and short-term responses. Cumulative effects affected 40.49% of vegetation, with a mean duration of 5.06 months. These findings provide a mechanistic basis for understanding drought impacts on vegetation WUE across contrasting frozen-soil environments.

## Highlights

• Vegetation water use efficiency declined with an increase–decrease–increase pattern
• A 0.6 gC m^−2^ mm^−1^ threshold separated the two frozen-soil types
• Response lags were longer in seasonally frozen soils than permafrost
• Drought accumulation affected 40.49% of vegetation for 5.06 months on average

## Graphical abstract

**Figure:**
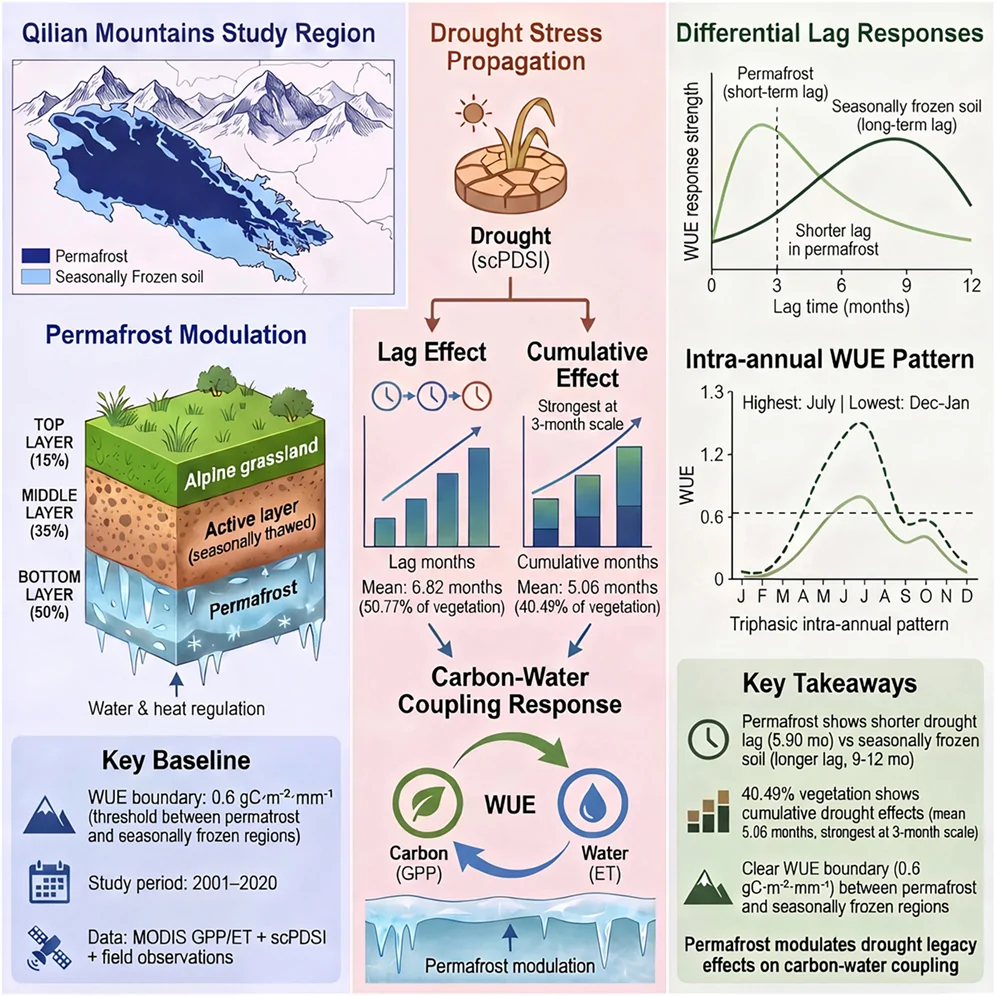

## Teaser

Earth-surface processes; Soil science; Environmental science; Global change; Hydrology; Soil hydrology

## Introduction

Frozen ground underlays extensive areas of the high latitudes and high-elevation areas at lower latitudes, falling into two major categories: seasonally frozen soil, and permafrost. In recent decades, air temperatures in high-latitude and high-altitude regions have risen faster than the global average.^1^ The temperature of the upper permafrost has risen by 0.5°C–2.0°C in the Northern Hemisphere,^2^ and rapid and extensive permafrost degradation will alter the hydrological and biogeochemical cycles in these regions.^3^ Moreover, the Permafrost, as an essential and important component of the Earth system, cannot be ignored and deeply involved in a range of geophysical processes, such as the global hydrological cycle,^4^ the carbon cycle,^5^ and ecological processes.^6^ Permafrost degradation alters the hydrothermal environment between vegetation and soil,^7^ affecting the availability of biogeochemical cycles and climate change during vegetation growth.

Vegetation plays a vital role in terrestrial ecosystems and energy balances, and its dynamics are closely related to climate change,^5^^,^^8^ affecting the carbon-water coupling and dynamic balance of ecosystems through photosynthesis and respiration.^9^^,^^10^ Water use efficiency (WUE), defined as the ratio of carbon uptake to water consumption, is an excellent indicator reflecting the integrated effects of carbon, energy, and water cycles on terrestrial ecosystem processes,^11^ and is widely used in vegetation dynamics studies.^12^^,^^13^ With global climate warming, the frozen regions are undergoing significant ecological transformations, which highlight the potentially significant impact of changes in water availability on vegetation dynamic processes and the carbon-water cycle.^6^^,^^7^ Therefore, elucidating the spatial-temporal patterns and drivers of WUE is essential to understanding the mechanism of change in water use efficiency (WUE) of frozen and permafrost state ecosystems in the context of climate change.^14^

Drought is widely recognized as the primary climate driver influencing vegetation growth and water-carbon coupling, particularly in climate changing^14^^,^^15^^,^. Drought effects on ecosystem WUE may vary with different dry or wet zones, drought severity, and vegetation type.^16^ Thus, understanding this multifaceted vulnerability is a prerequisite for predicting the damage in ecosystems facing future drought events.^10^^,^^17^ The Palmer Drought Severity Index (PDSI) and self-calibrated PDSI (sc_PDSI) are commonly used to quantify drought on a single timescale,^18^^,^^19^ involving temperature and soil moisture in addition to precipitation.^10^^,^^17^ Generally, the extent to which vegetation growth responds to climate extremes by coupling with carbon and water, and threshold ranges, is determined by analyzing the correlation between climate extremes(e.g., sc_SPEI) and changes in vegetation ecological indices(e.g., WUE).^20^^,^^21^ Therefore, quantifying the vegetation response to drought is crucial for understanding vegetation-drought coupling and the vulnerability of the ecological environment to climate change, especially in the area of the active layer and permafrost.^5^^,^^8^

Temporal effects are a non-negligible phenomenon in climate-vegetation interactions, including time lag and accumulation.^8^ Generally, the duration of the effects of drought on vegetation varies from a few hours to several years.^8^^,^^22^ The intensity and duration of drought events have a stronger impact on vegetation production patterns with a carbon-water coupling mechanism,^8^^,^^22^ and this long-term effect can be divided into lag effect and cumulative effect.^21^^,^^22^ Some studies have shown that the correlation analysis of the time-lag effect and the time-cumulative effect of vegetation and climate drought has found that the cumulative climate effect within one month and a few months ago is more sensitive.^23^ The lack of considering extreme climate and time-lag and accumulation effects may lead to an underestimation of the effect of drought on vegetation dynamics. Therefore, a significant gap between quantifying drought propagation thresholds and effectively implementing drought early warning systems and interrupting drought propagation.

The Qilian Mountain is located in the northeastern part of the Tibetan Plateau, spanning across Gansu and Qinghai provinces (Figure 1). They are the source and water supply area for the inland river basins of the Hexi Corridor and are one of China’s important ecological barrier zones.^24^^,^^25^ The well-developed permafrost and its preservation conditions have created a unique ecological balance and material cycling, making it a typical distribution area for high-mountain permafrost in Northwest China. Changes in the thickness of the active layer in permafrost regions directly affect the soil’s hydrothermal properties and other physical characteristics, thereby influencing the hydrothermal exchange and carbon cycling between the land and atmosphere, causing changes in the surface vegetation ecosystem, and even affecting the local climate of the permafrost region and the global climate.^26^^,^^27^ Seasonally frozen soil has the characteristic of unidirectional freezing and bidirectional thawing,^28^ and the hydrothermal coupling characteristics of the soil are important reasons for the differences in vegetation types, cover, carbon storage, and allocation.^29^ However, studies on the spatiotemporal distribution of WUE and its response to drought in high-latitude alpine permafrost regions^28^^,^^30^ have not yet clarified the spatiotemporal variation characteristics of the vegetation carbon-water coupling relationship under the influence of spatial differentiation between permafrost and seasonally frozen soil regions.

**Figure 1:**
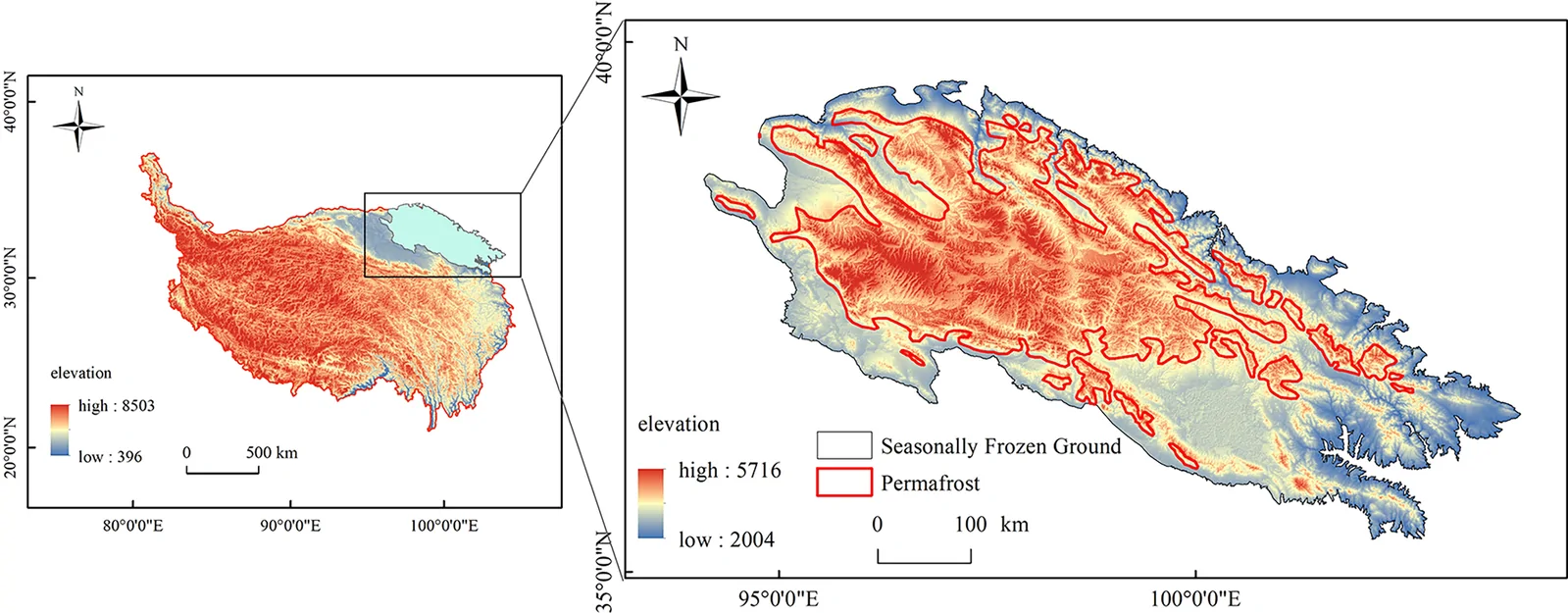
Geographical distribution of the study area The left image shows the Tibetan Plateau, and the right image shows the study area, the Qilian Mountains.

The objectives of this study are as follows: (1) to reveal the spatial-temporal variations of WUE in permafrost and frozen soil areas, (2) to explore the time-lagged and cumulative effects of drought on WUE in alpine regions (3) to analyze the relationships among the permafrost and frozen ground changes, vegetation growth, and sc_PDSI in the Qilian Mountains from 2001 to 2020.

## Results

### Spatial-temporal patterns of WUE

#### Spatial characteristics of WUE

From the monthly variation perspective (Figure 2), the average WUE of the Qilian Mountains shows a trend of first increasing, then decreasing, with the highest value in July (1.57 gC m^−2^ mm^−1^) and the lowest value in December (0.001 gC m^−2^ mm^−1^). High WUE values are predominantly located in seasonal permafrost regions. Over the past 20 years, the average WUE of vegetation in the Qilian Mountains is 0.56 gC m^−2^ mm^−1^, exhibiting a spatial distribution pattern of higher values in the southeast and lower values in the northwest. In the study area, 75.7% of the vegetation has a WUE lower than 0.8 gC m^−2^ mm^−1^, and 34.8% has a WUE lower than 0.4 gC m^−2^ mm^−1^, mainly distributed in high mountain areas with perennial permafrost, such as Har Lake, Chaidamu Mountains, Shule Nan Mountains, Datong Mountains, Lenglong Ridge, and Tuolai Mountains (Figure 3). Conversely, 24.2% of the vegetation has a WUE higher than 0.8 gC m^−2^ mm^−1^, primarily located in seasonal permafrost regions around Qinghai Lake, the Huangshui River Basin, the Heihe River Basin, and near the Wushao Ridge.

**Figure 2:**
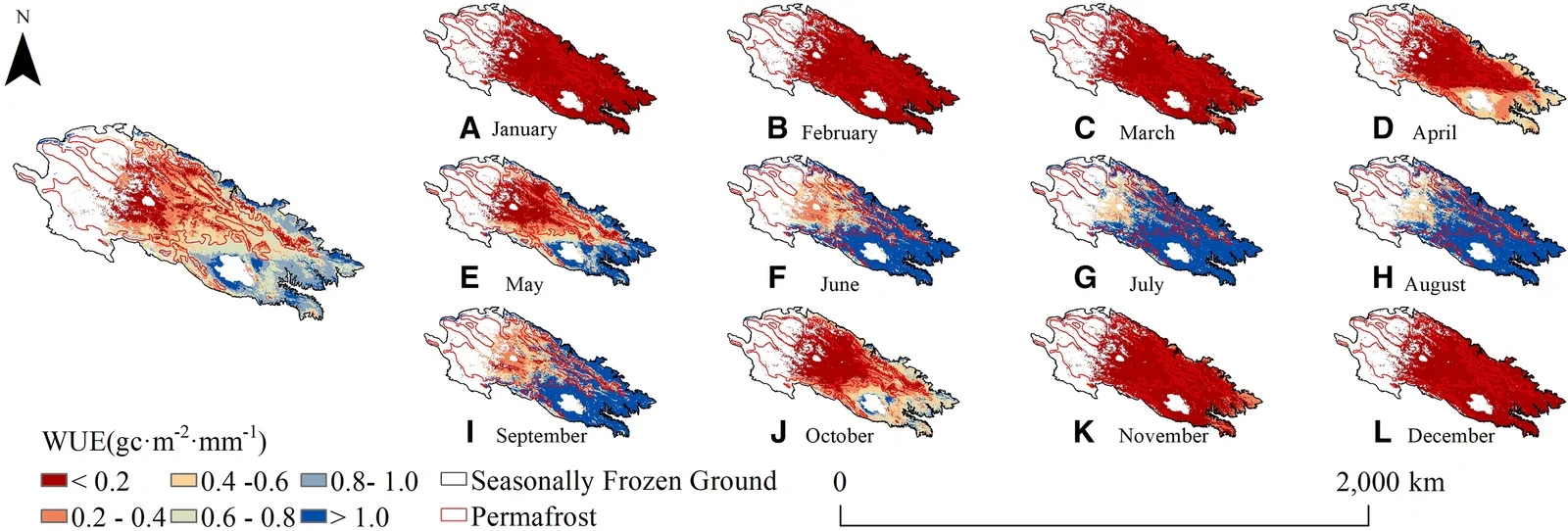
Spatial patterns of the Qilian Mountains from 2001 to 2020 and monthly spatial patterns The left image shows the spatial pattern of the Qilian Mountains from 2001 to 2020; The right images (A–L) show the monthly spatial patterns from 2001 to 2020.

**Figure 3:**
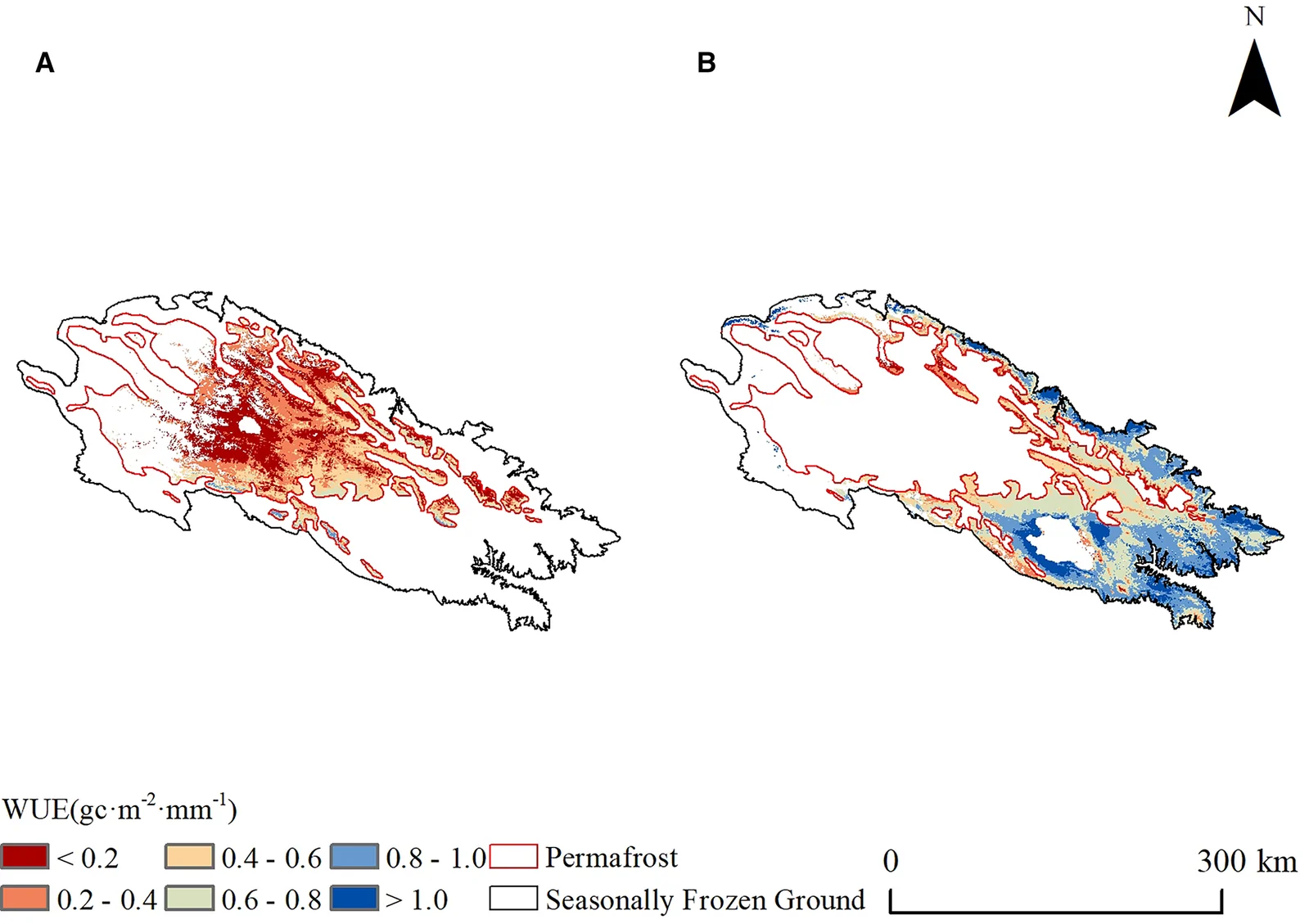
Water use efficiency in the permafrost (A) and seasonally frozen soil (B) regions, Qilian Mountains, 2001–2020

#### Temporal characteristics of WUE

From the monthly changes in WUE, it is observed that the WUE in the Qilian Mountains shows an increasing trend in January, March, and October to December, with the most significant increase occurring in November. The area with increasing WUE accounts for 95.79% of the total area, among which the area with a significant increase accounts for 42.51% of the total area. Conversely, a decreasing trend is observed in February and from April to August, with the most significant decrease occurring in June. The area with decreasing WUE accounts for 97.17% of the total area, among which the area with a significant decrease accounts for 57.91% of the total area (Figure 4). From 2001 to 2020, the overall trend of WUE in the Qilian Mountains region is a slow decline (Figure 5). The highest value of WUE in the past 20 years was recorded in June 2001, at 2.1 gC·m^−2^ ·mm^−1^. The WUE reaches its lowest value from December to February of the following year, almost approaching zero, while the highest values are observed from July to August each year. By comparing the interannual changes in WUE between the permafrost and seasonally frozen soil areas of the Qilian Mountains, it is found that the changes in WUE in both areas are similar to the overall trend of the study area, showing a slow decline (Figure 5). The correlation of WUE between the study area and the seasonally frozen soil area (*R* = 0.9968) is stronger than that between the study area and the permafrost area (*R* = 0.9803) (Figure 6). The small difference in area between the permafrost (47.51%) and seasonally frozen soil (52.49%) areas indicates that the changes in WUE in the Qilian Mountains are primarily determined by the seasonally frozen soil. Comparing the monthly changes in WUE between the permafrost and seasonally frozen soil areas, it is observed that the changes in the seasonally frozen soil area are consistent with the overall trend of the Qilian Mountains. However, in the permafrost area, WUE is almost zero in January, February, and December due to snow and ice cover. From March to September, WUE in the permafrost area shows a decreasing trend, with the most significant decrease occurring in June. The area with a decreasing trend accounts for 97.38% of the total area, among which the area with a significant decrease accounts for 35.70% of the total area. From October to November, an increa sing trend is observed, with the most significant increase occurring in November. The area with an increasing trend accounts for 95.79% of the total area, among which the area with a significant increase account for 42.51% of the total area.

**Figure 4:**
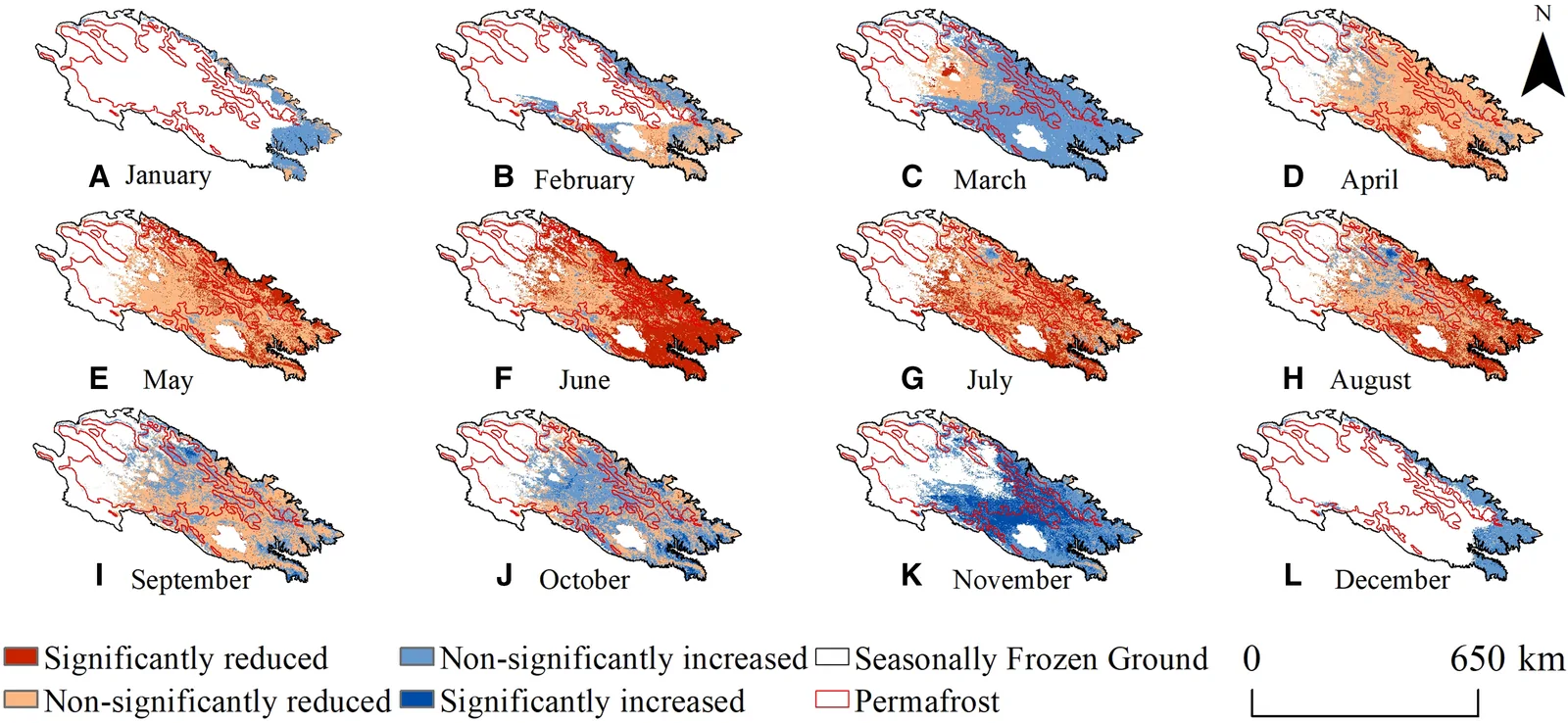
Monthly trends of WUE in the Qilian Mountains from 2001 to 2020 (A–L) Show the monthly variation trends of the Qilian Mountains from 2001 to 2020.

**Figure 5:**
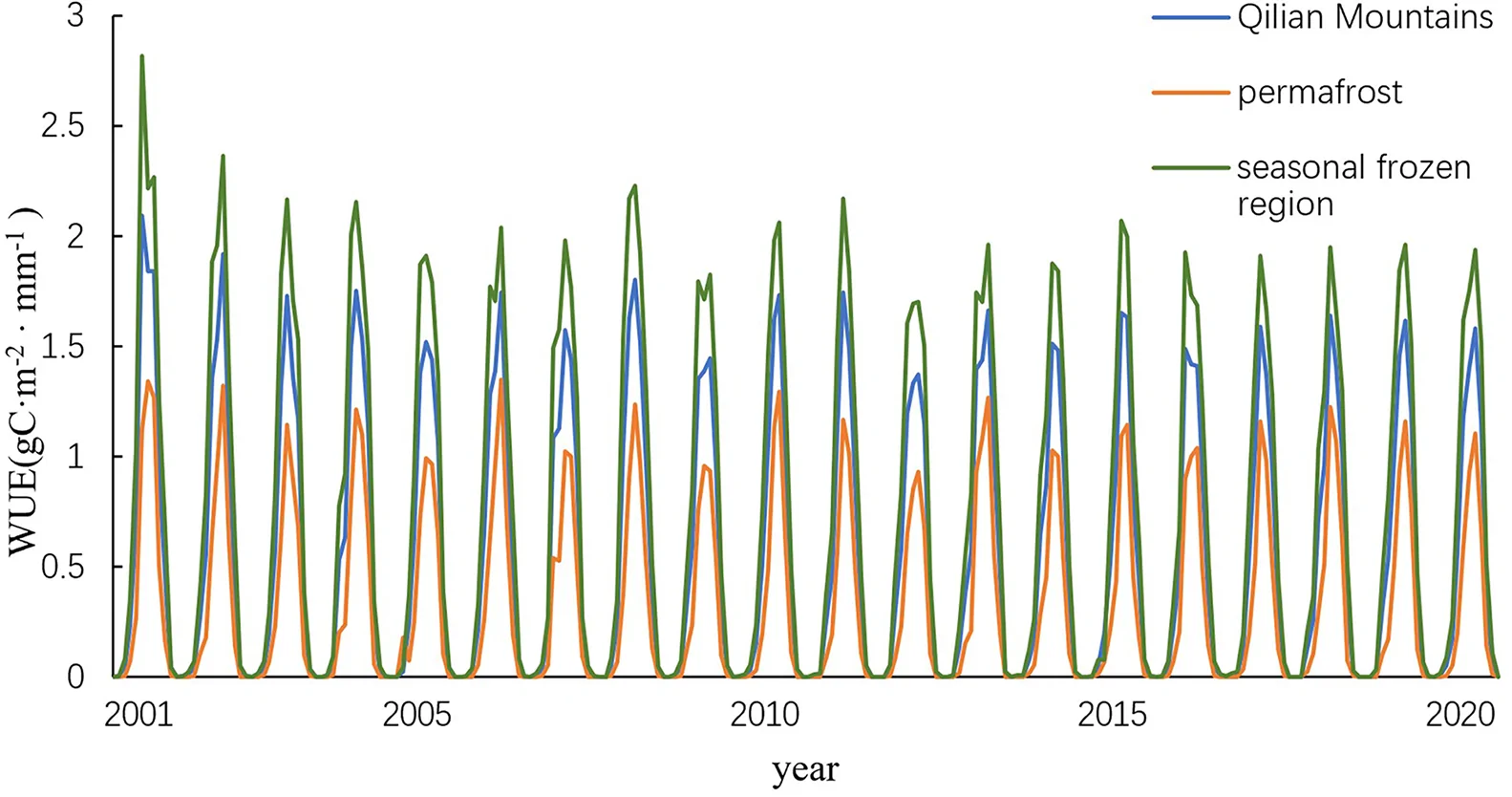
Monthly changes of WUE in the Qilian Mountains region from 2001 to 2020 Lines represent the monthly spatially averaged WUE values for the Qilian Mountains, seasonally frozen soil area, and permafrost area from 2001 to 2020. Each value was calculated using all valid pixels within the corresponding region.

**Figure 6:**
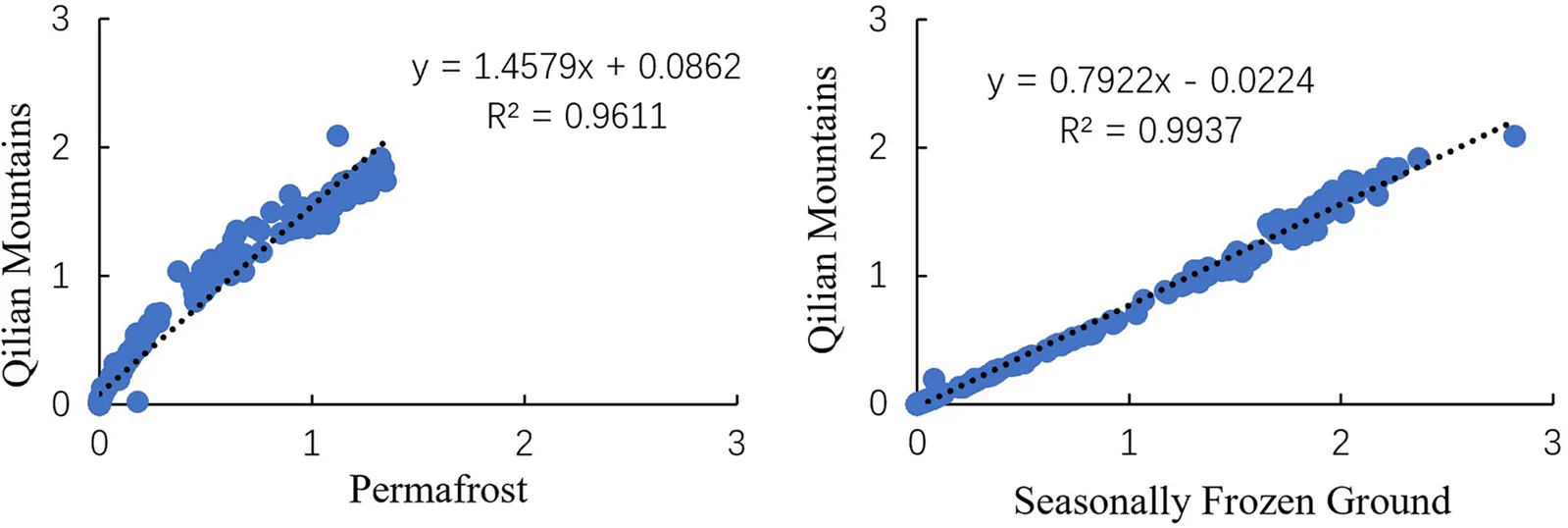
Correlation of WUE between the permafrost areas and seasonally frozen soil areas Each point represents one monthly regional mean WUE value. Monthly regional means were calculated from all valid vegetation pixels within the corresponding region. Pearson’s correlation coefficients were calculated using the monthly time series.

### Lagged and cumulative effects of scPDSI on WUE

#### Lagged effect of scPDSI on WUE

Based on the correlation analysis between WUE and the interpolated scPDSI data (Figure 7) with different lag months in the Qilian Mountains, the maximum correlation coefficient (Rmax-lag) for the lagged effect of scPDSI on WUE was obtained. The correlation between scPDSI and WUE in the Qilian Mountains region is weak, with a correlation coefficient of −0.16. Given the large area of permafrost in the region, which can supply moisture to vegetation under drought conditions, it can be inferred that the lagged effect of scPDSI has a minimal impact on WUE in the Qilian Mountains. In terms of the significance of the maximum correlation coefficient for the lagged effect, the region mainly shows extremely significant negative correlation, with 50.71% of the total vegetation area in the Qilian Mountains exhibiting this relationship. Comparing the permafrost and seasonally frozen soil areas within the Qilian Mountains, both show extremely significant negative correlation between WUE and scPDSI with different lag months. The permafrost area has an extremely significant negative correlation covering 64.96% of its total vegetation area, while the seasonally frozen soil area has 40.07%. In addition to these negative correlations, large areas of extremely significant positive correlation also appear in both regions. In the permafrost area, this positive correlation is mainly concentrated near Har Lake, covering 19.06% of the total vegetation area. In the seasonally frozen soil area, it is mainly concentrated near Qinghai Lake, covering 34.06% of the total vegetation area.

**Figure 7:**
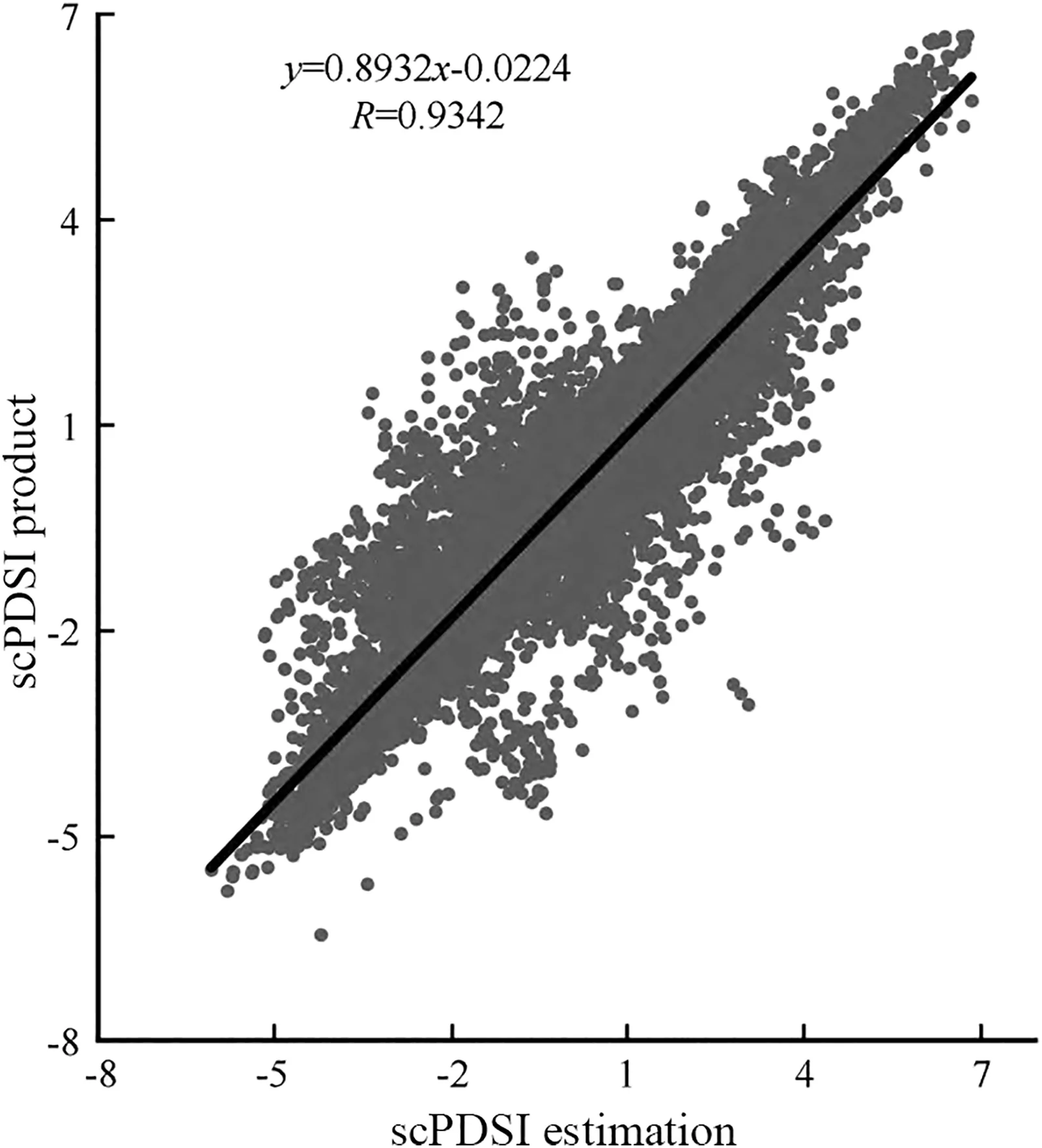
Accuracy validation of the scPDSI data product Twenty percent of the original scPDSI grid cells were randomly selected as validation samples before spatial interpolation. Each point represents a comparison between the original scPDSI value and the corresponding value estimated by Anusplin 4.2. The solid line represents the linear regression fit. The regression equation and correlation coefficient (R) are shown in the figure.

From the spatial distribution characteristics of the maximum lag months of WUE and scPDSI (Figure 8), it can be seen that 50.77% of the vegetation in the Qilian Mountains region shows a lagged effect on drought (1≤lag month≤12), with an average lag of 6.82 months. The response of WUE to scPDSI mainly includes no lag effect (lag month = 0), short-term lag (1≤lag month≤4), and long-term lag (9≤lag month≤12). Short-term lag and no lag are mainly distributed in the permafrost area, while long-term lag is mainly found in the seasonally frozen soil area. To further analyze the lagged effect of drought on permafrost and seasonally frozen soil areas, the average lag months were statistically analyzed separately for these two regions. In terms of the lagged effect of scPDSI, the seasonally frozen soil area has a higher average lag of 7.24 months compared to the overall average of the Qilian Mountains, while the permafrost area has a lower average lag of 5.90 months. The response of WUE to scPDSI in the seasonally frozen soil area is mainly characterized by no lag and long-term lag, while in the permafrost area, it is mainly no lag and short-term lag. Comparing the correlation of the lagged effect of scPDSI on WUE (Figure 9) with the distribution of maximum lag months, it was found that in areas with a lagged effect, scPDSI shows a positive correlation with WUE. In the permafrost area, short-term and medium-term lags show an extremely significant positive correlation, while the long-term lag shows a significant positive correlation. In the seasonally frozen soil area, medium-term and long-term lags show an extremely significant positive correlation, while the short-term lag shows an extremely significant positive correlation near Wushao Ridge.

**Figure 8:**
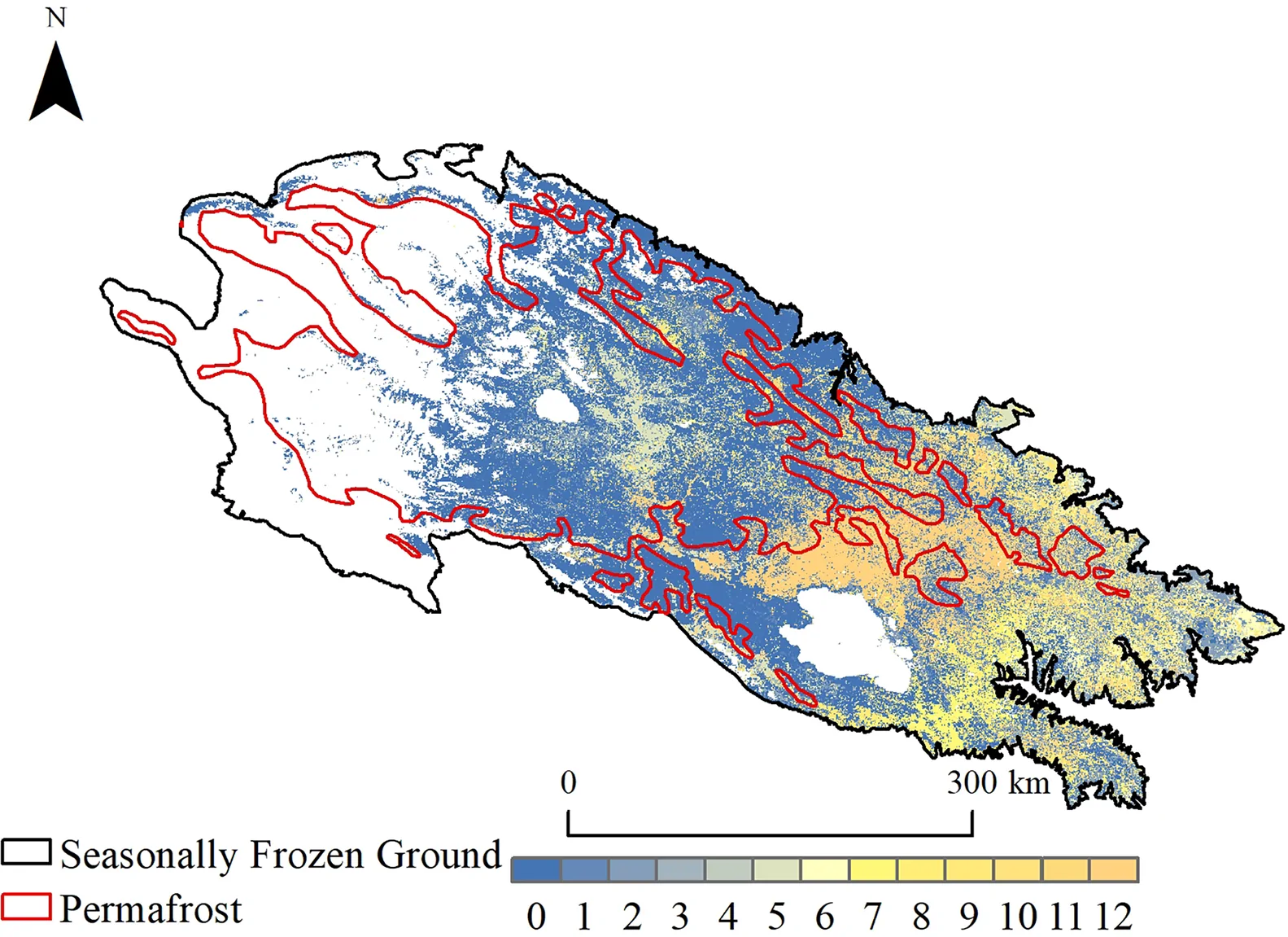
Spatial distribution of maximum lag months between WUE and the scPDSI in the Qilian Mountains Region

**Figure 9:**
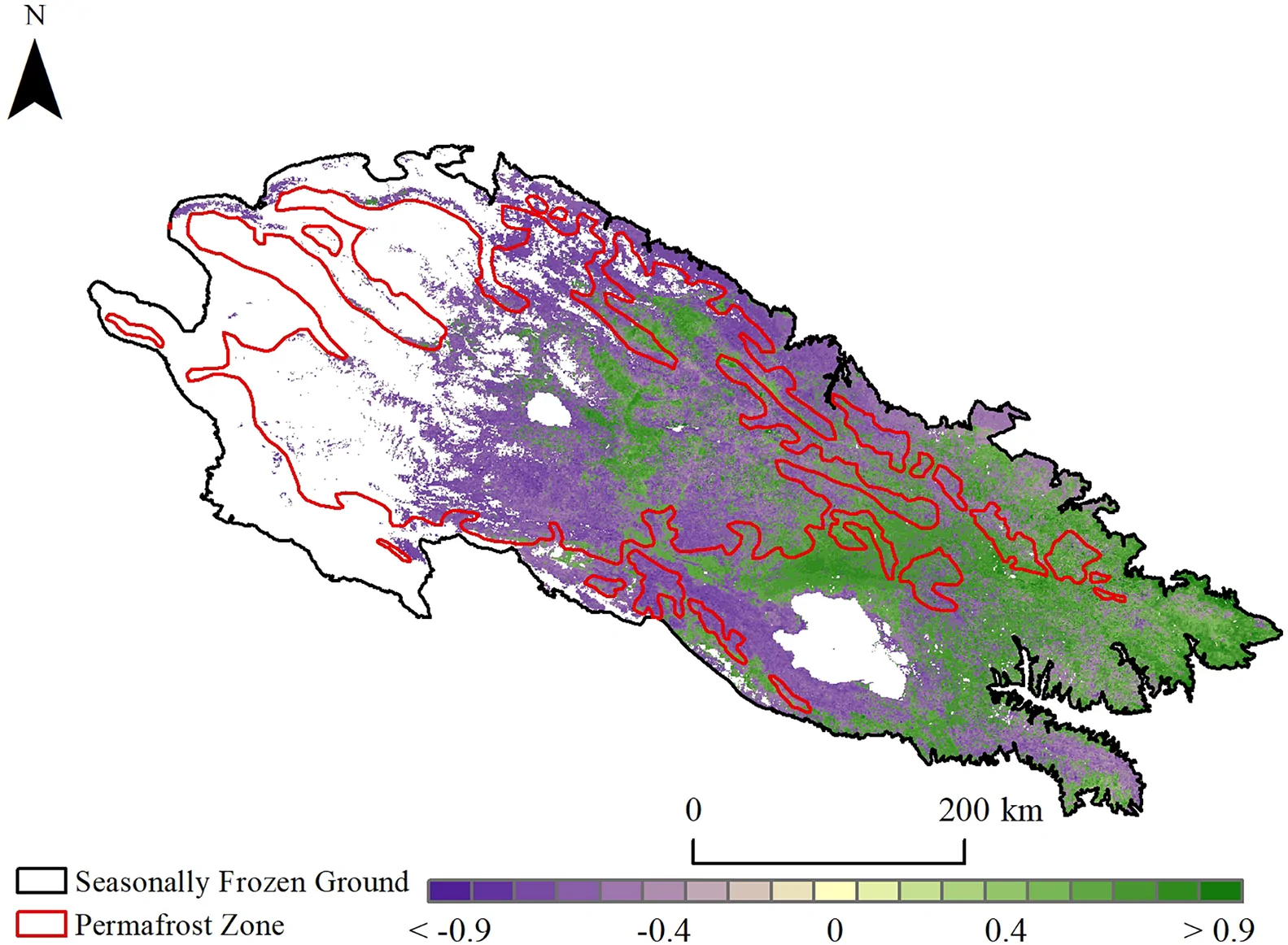
Correlation of the lagged effect of scPDSI on WUE in the Qilian Mountains region

#### Cumulative effect of scPDSI on WUE

Hydrological conditions at different times can either promote or inhibit the carbon-water conversion of vegetation, and these conditions act on the water use efficiency (WUE) of the current growth stage of vegetation. The combined effects of these factors form the cumulative effect on the water use efficiency of vegetation in the next stage. Analysis of the maximum correlation coefficient (Rmax-acc) between scPDSI and WUE at different accumulation scales revealed that, similar to the lag effect, the correlation between scPDSI and WUE in the Qilian Mountains region is weak across different accumulation scales, with a correlation coefficient of −0.39. In terms of the significance of the correlation coefficient, the region mainly shows an extremely significant negative correlation, with 54.01% of the vegetation area in the Qilian Mountains showing an extremely significant negative correlation. Comparing the spatial distribution of the correlation coefficients between WUE and scPDSI for different accumulation months in permafrost and seasonally frozen soil areas, it was found that the distribution of significant correlation coefficients in both areas is the same as that of the Qilian Mountains as a whole, with extremely significant negative correlation. The area with extremely significant negative correlation in permafrost areas accounts for 68.87% of the total vegetation area, while in seasonally frozen soil areas, it accounts for 42.92%. Similar to the lag effect, large areas of extremely significant positive correlation also appear in both permafrost and seasonally frozen soil areas. The extremely significant positive correlation in permafrost areas is mainly concentrated near the Shule Nan Mountains, while in seasonally frozen soil areas, it is mainly concentrated near Xining City.

From the spatial distribution characteristics of the maximum accumulation months of WUE and scPDSI (Figure 10), it can be seen that 40.49% of the vegetation in the Qilian Mountains region shows a cumulative effect on drought (1≤accumulation month≤12), with an average accumulation month of 5.06. This indicates that the WUE in the study area exhibits a certain degree of cumulative effect. The response of WUE to scPDSI is different from the lag effect, mainly showing no cumulative effect (accumulation month = 0) and short-term accumulation (1≤accumulation month≤4), with short-term accumulation mainly distributed near Har Lake. Further statistics on the average accumulation months in permafrost and seasonally frozen soil areas show that the accumulation months in seasonally frozen soil areas are higher than the overall average in the Qilian Mountains, with an average of 5.88 months. In contrast, the accumulation months in permafrost areas are lower than the overall average, with an average of 3.88 months. Seasonally frozen soil areas mainly show no cumulative effect and short-term accumulation, with large areas of long-term accumulation appearing near Xining City. Similar to seasonally frozen soil areas, permafrost areas mainly show no cumulative and short-term accumulation effects. Comparing the correlation of the cumulative effect of scPDSI on WUE and the maximum accumulation months in the Qilian Mountains region (Figure 11), it was found that the mid-term accumulation in permafrost areas shows an extremely significant positive correlation. In seasonally frozen soil areas, the cumulative effect of scPDSI on WUE shows a positive correlation near Wushao Ridge.

**Figure 10:**
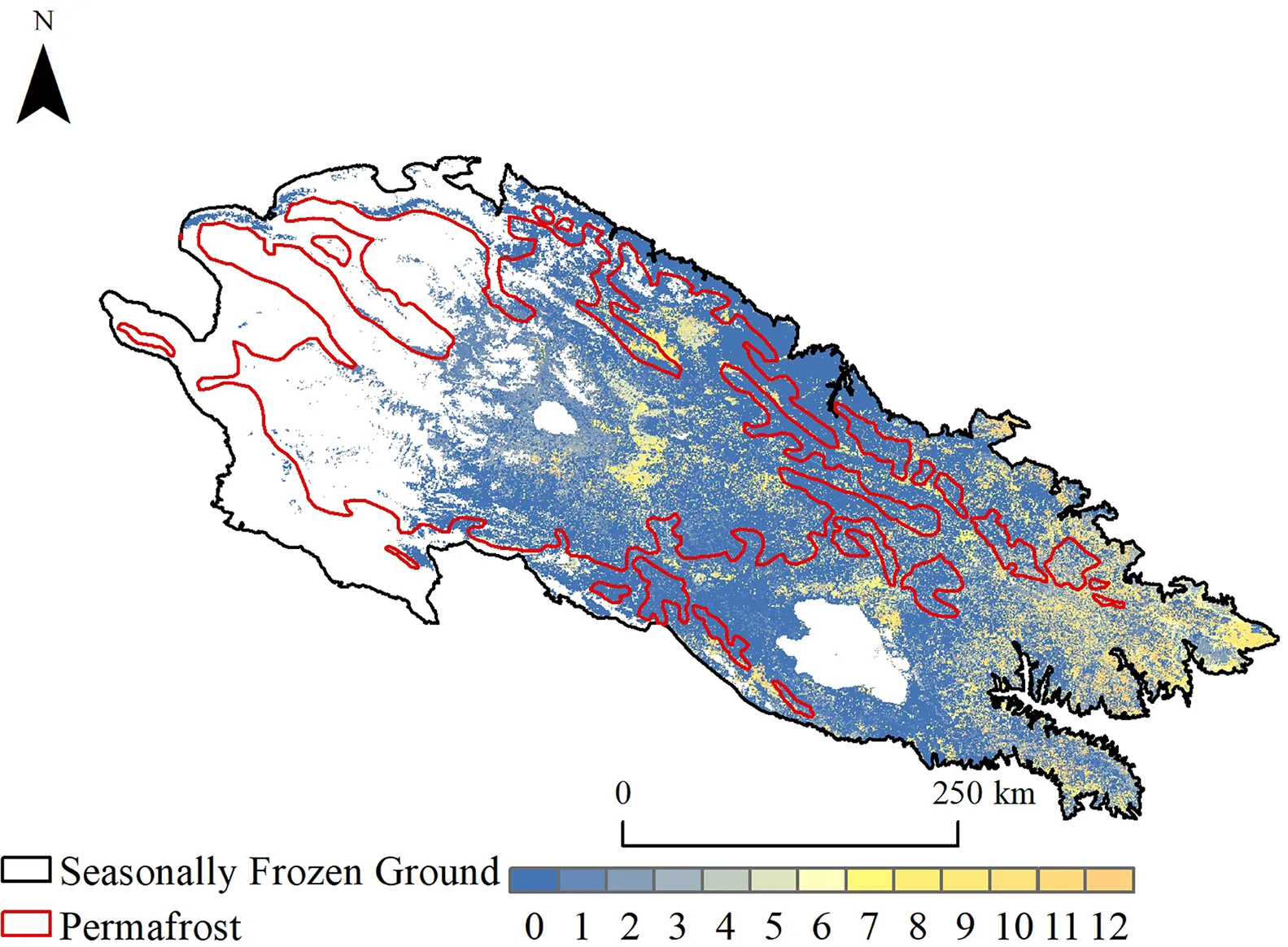
Distribution of the maximum cumulative months of WUE and scPDSI in the Qilian Mountains Region

**Figure 11:**
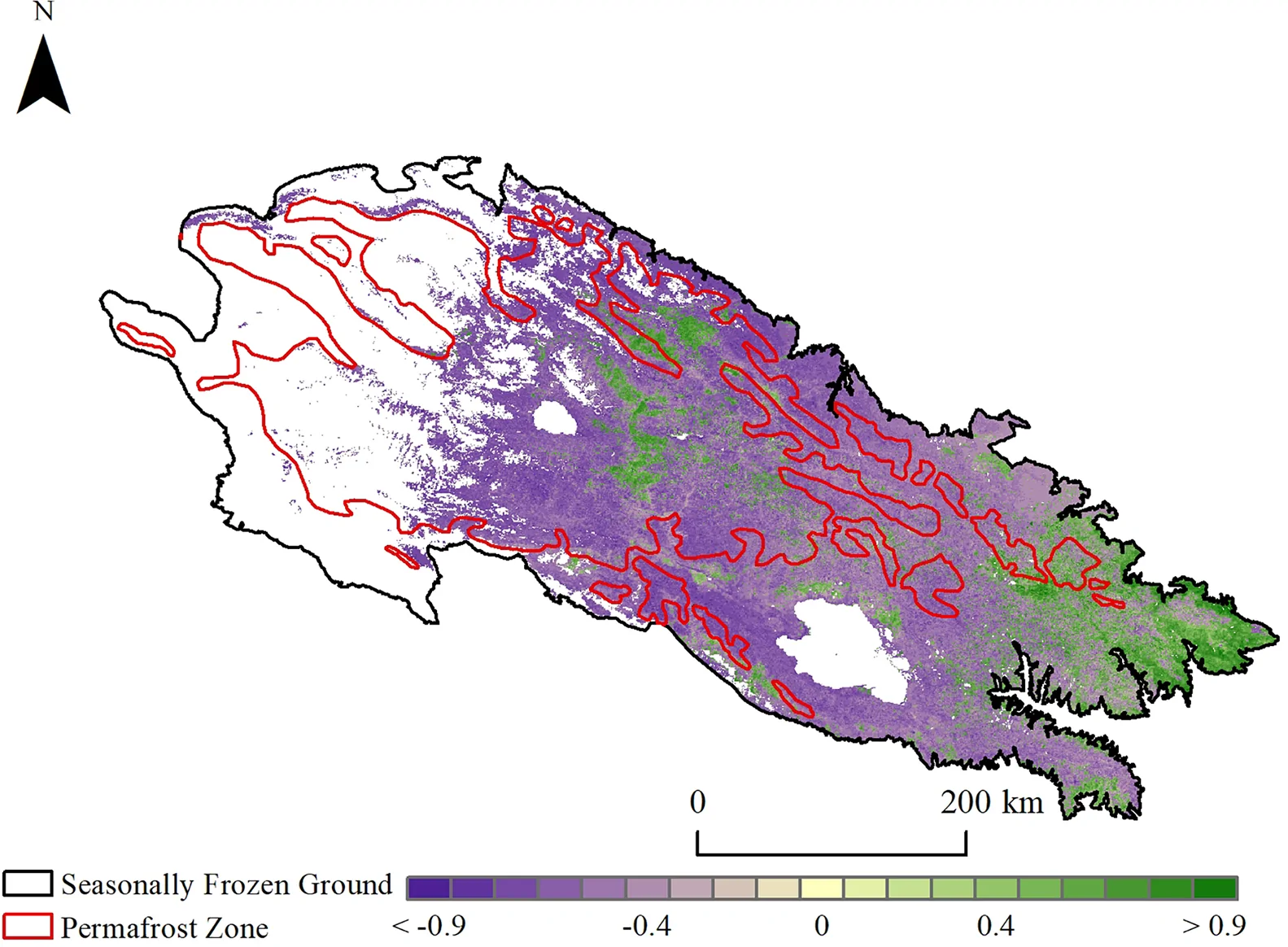
Correlation between scPDSI and the cumulative effect of WUE in the Qilian Mountains Region

## Discussion

### Spatial-temporal changes in WUE from 2001 to 2020

The Qilian Mountains, serving as the boundary between the three major geomorphological units of the Tibetan Plateau, the Mongolian Plateau, and the Loess Plateau,^31^ exhibit distinct spatial distribution characteristics of WUE due to their diverse topography and varying hydrothermal conditions.^32^ In the southeastern part, abundant vegetation cover and a well-balanced hydrological system maintain high soil moisture, providing a favorable basis for carbon sequestration by vegetation, resulting in higher WUE values. In contrast, the northwestern region, with sparse vegetation and unstable hydrological conditions, has lower WUE values. The WUE in seasonally frozen soil areas is generally higher than that in permafrost regions. This is attributed to the good vegetation cover and diverse types in seasonally frozen soil areas, which effectively enhance the carbon sequestration capacity per unit area. Additionally, abundant meltwater and precipitation promote vegetation transpiration, increasing water consumption. Conversely, permafrost areas are predominantly characterized by alpine wastelands and alpine meadows with poor vegetation cover. The fragile ecological environment results in unstable carbon sequestration capacity, and the long-term frozen state restricts vegetation transpiration, leading to relatively lower WUE values. From 2001 to 2020, the trend in WUE in the Qilian Mountains was mainly influenced by seasonally frozen soil areas. Between October and January of the following year, the incomplete freezing state of seasonally frozen soil provides ample moisture for the soil.^27^ Suitable moisture conditions and lower temperatures promote the growth of alpine meadows, thereby increasing vegetation carbon sequestration and leading to an upward trend in WUE. In February, human activities, such as the expansion of cultivated land, alter the growth environment of vegetation. The earlier recovery of vegetation promotes growth, and WUE continues to rise. However, from April to September, the WUE in the Qilian Mountains shows a downward trend in both seasonally frozen and permafrost regions. The accumulation of spring meltwater and increased rainfall raises soil moisture content. However, due to the slow increase in temperature in alpine meadow areas, the vegetation’s carbon sequestration capacity does not significantly improve, negatively affecting vegetation productivity.^33^ This results in a decrease in the amount of organic matter fixed per unit of water, leading to a decline in WUE. In June, the accumulation of meltwater from the spring thaw reaches its peak; combined with higher temperatures, excessive moisture inhibits vegetation growth, and the decline in WUE is most pronounced. By November, the amount of meltwater in the soil freeze-thaw cycle exceeds the amount of frozen water. The improvement in alpine meadow vegetation cover increases vegetation carbon sequestration, and WUE significantly rises.^34^ In summary, the temporal dynamics of WUE further revealed that WUE in the Qilian Mountains showed an increasing trend in January, March, and from October to December, with the most significant increase in November. It showed a decreasing trend in February and April-August, and reached the most significant reduction in June. The changes in the seasonally frozen soil areas were consistent with the overall trend of the Qilian Mountains. In the permafrost areas, WUE is close to zero in January-February and December, showing a decreasing trend from March to September, with the most significant decrease in June, and an increasing trend from October to November, with the most significant increase in November.

### Lagged effect on WUE from 2001 to 2020

The analysis of the lag effect between WUE and scPDSI showed that 50.77% of the vegetation in the Qilian Mountain region had a lag effect on drought (Figure 12), with the average lag month of 6.82 months. This result indicates that the vegetation response to drought is not immediate but gradually manifests several months after the drought conditions occur. Vegetation in the seasonally frozen soil areas responds relatively quickly to drought, with an average lag of 7.24 months. This may be due to the higher soil temperatures and more active water cycling in this region, which provide faster water replenishment. According to the lagged types in the Qilian Mountains (Figure 13), vegetation in the seasonally frozen soil areas has a better adaptation to the water fluctuations caused by drought and can tolerate a certain period of water shortage.^35^ This is likely because the southeastern part of the region has undergone long-term vegetation ecological restoration, with vegetation showing medium to high coverage. This situation results in a predominantly medium-term lag in this area. In the seasonally frozen soil areas north of Qinghai Lake, the presence of marshy terrain with abundant free water surfaces provides sufficient water supply for the early growth of local vegetation. However, in the later stages of vegetation growth, other limitations such as nitrogen and sunlight affect vegetation growth,^36^^,^^37^ leading to the local vegetation exhibiting long-term lag effects. Vegetation in the permafrost areas shows a significant lagged response to drought, with an average lag of 5.90 months. This may be related to the low soil temperatures, thin active layers, and short growing seasons in this region. Compared with seasonally frozen soil areas, the temperature rise caused by drought in permafrost areas leads to an increase in heat, which delays the vegetation response to drought due to the water supply from the melting permafrost layer. Although short-term permafrost meltwater provides some water supply for vegetation, drought conditions in the permafrost areas are also affected by rising temperatures and soil water loss. Therefore, even with meltwater, vegetation may still experience water shortages, and the rapid response of vegetation to the water supply from permafrost meltwater may change with permafrost degradation, leading to water constraints that affect vegetation survival.^38^ As a result, no lag and short-term lag may be more common for vegetation in the permafrost areas. Land use and land cover change (LUCC) also have a significant impact on the lagged effect of WUE. Human activities such as urban expansion, agricultural cultivation, and deforestation can alter the surface energy and water balance, thereby affecting the water use efficiency of vegetation. Vegetation restoration and ecological restoration projects improve soil water retention capacity and enhance vegetation’s adaptability to drought. In summary, the correlation between scPDSI and WUE for different lagged months in the Qilian Mountains was weak, with a correlation coefficient of −0.16. The lagged effect of scPDSI on WUE in the Qilian Mountains mainly showed an extremely significant negative correlation. This was the same for both the permafrost and seasonally frozen soil areas. Comparing the correlation between the lagged effect of scPDSI on WUE and the distribution of the maximum lagged months, it was found that in areas with a lagged effect, scPDSI showed a positive correlation with WUE.

**Figure 12:**
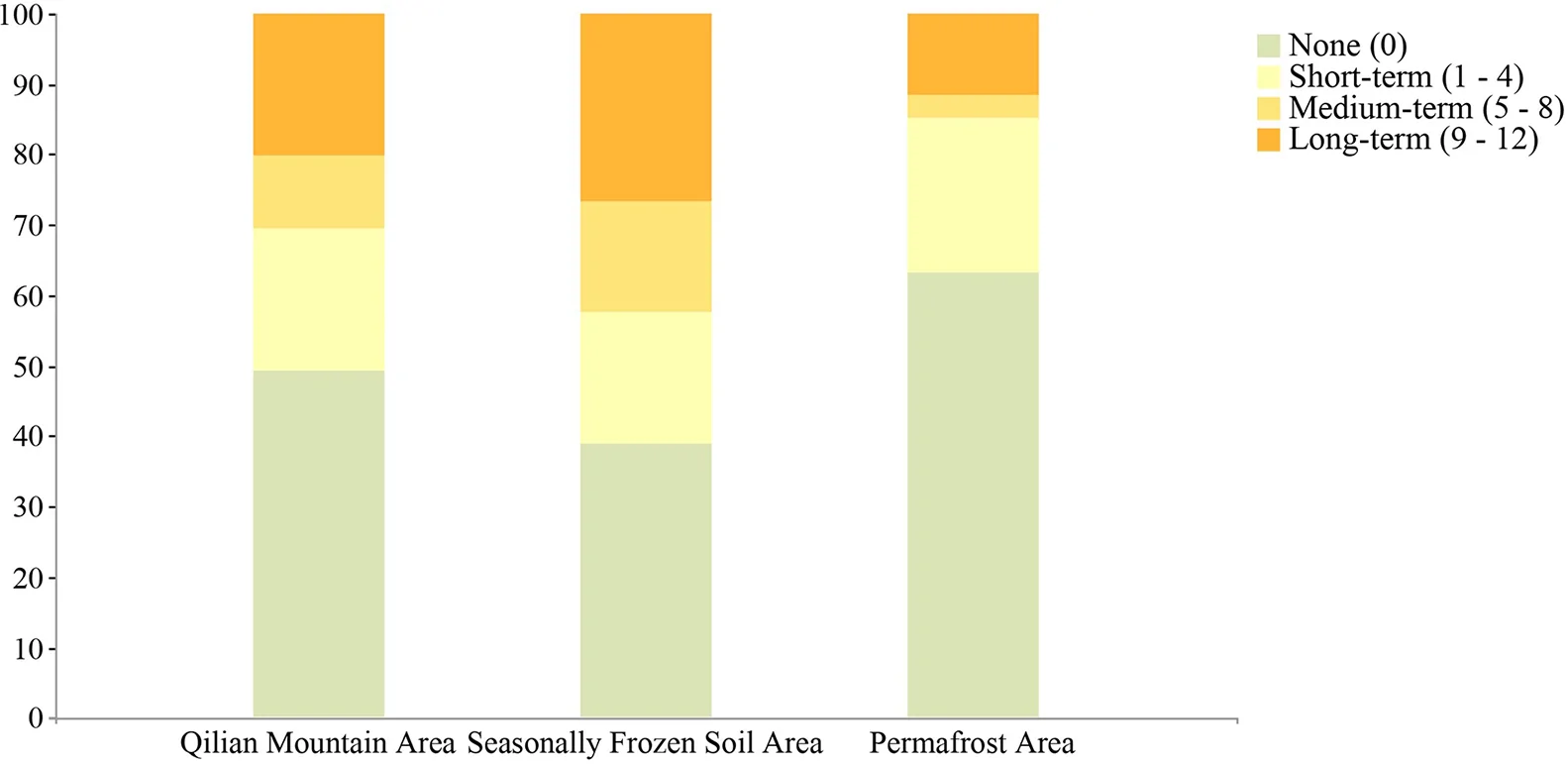
Percentage of lagged types Bars show the percentage distribution of lagged-response types within the Qilian Mountains, seasonally frozen soil area, and permafrost area. Percentages were calculated as the proportion of valid vegetation pixels assigned to each lag category within the corresponding region. Each bar sums to 100%.

**Figure 13:**
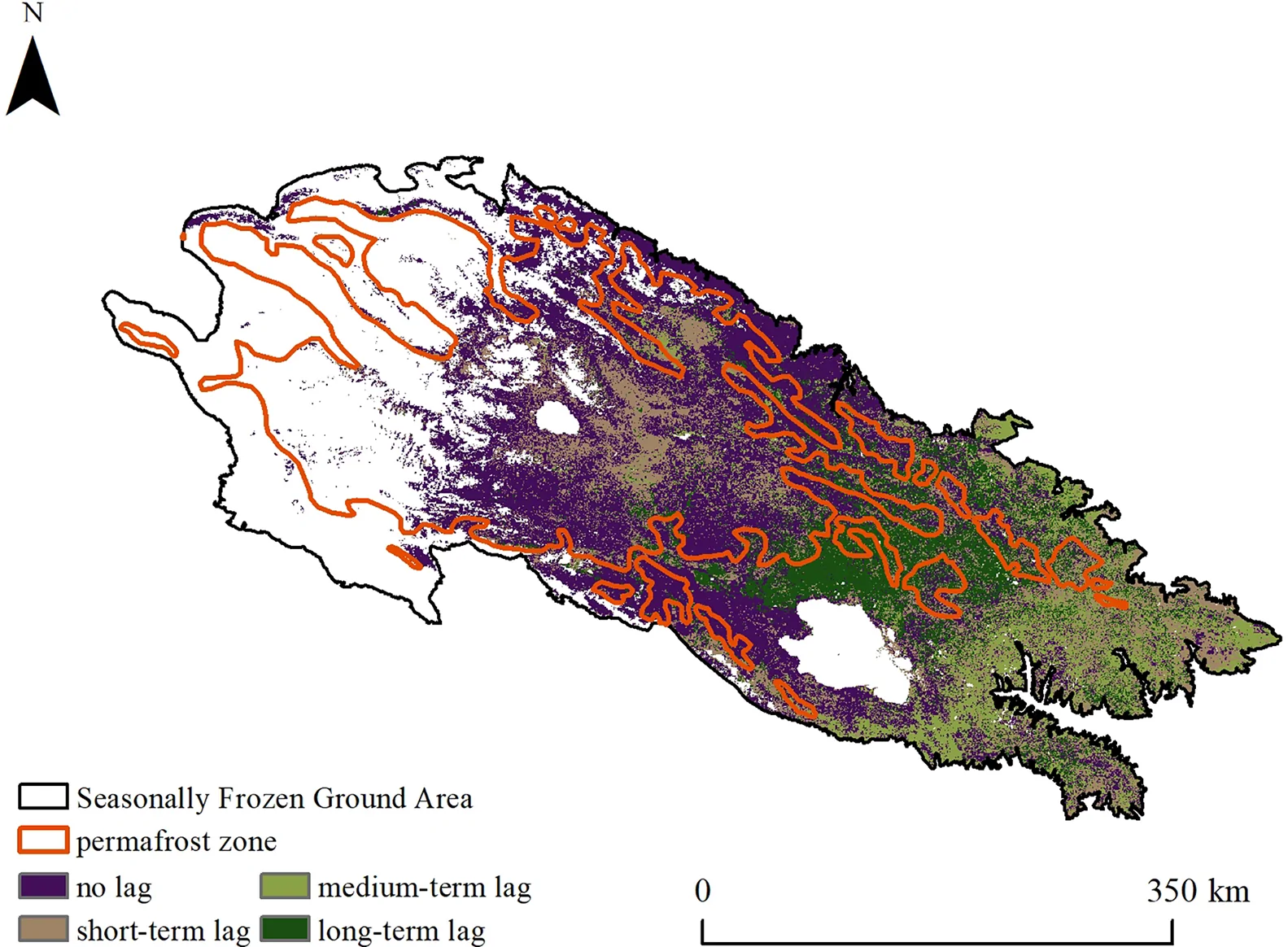
Lagged types in the Qilian Mountains

### Cumulative effects of WUE from 2001 to 2020

The analysis of the cumulative effect of WUE and scPDSI showed that 40.49% of the vegetation had a cumulative effect on drought (Figure 14), with a mean cumulative month of 5.06 months. This finding reveals the persistence of WUE response to drought conditions, meaning that drought conditions in preceding months significantly impact the WUE of the current month. According to the land type distribution in the Qilian Mountains (Figure 15), the vegetation in the permafrost areas of the study region is primarily alpine meadow. In high-altitude regions, alpine meadows increase their resistance to environmental stress with increasing altitude.^39^ Moreover, the depth of the permafrost layer is inversely proportional to altitude; the higher the altitude, the shallower the permafrost layer. Therefore, in high-altitude areas, alpine meadows can benefit from the water supply provided by the melting of permafrost. As a result, drought requires a longer cumulative period to affect vegetation growth in these high-altitude regions. However, the amount of meltwater from the permafrost layer in the permafrost areas is limited and cannot continuously provide a water supply to alpine meadows. Additionally, the root systems of alpine meadows are relatively shallow and can only absorb moisture from the surface soil.^8^ This means that alpine meadows can only strongly resist the cumulative effects of drought in the short term, which is also the reason why some parts of the region exhibit short-term and medium-term cumulative effects. According to the cumulative types in the Qilian Mountains (Figure 16), compared with the permafrost areas, the vegetation in the eastern part of the seasonally frozen soil areas is mostly forest, with well-developed root systems and stronger water retention capacity than alpine meadows. This also helps to reduce soil erosion, resulting in higher soil moisture content in the area. This situation enables vegetation to have stronger resistance to the cumulative effects of drought, mostly showing medium- and long-term cumulative effects. The correlation between scPDSI and WUE in the Qilian Mountains was weak, with a correlation coefficient of −0.39. The significance of the cumulative effect of scPDSI on WUE in the overall Qilian Mountains, permafrost areas, and seasonally frozen soil areas is consistent with the lagged effect. Comparing the correlation of the cumulative effect of scPDSI on WUE and the maximum cumulative monthly distribution, the permafrost area of the Qilian Mountains exhibited the strongest positive correlation between scPDSI and WUE across cumulative months.

**Figure 14:**
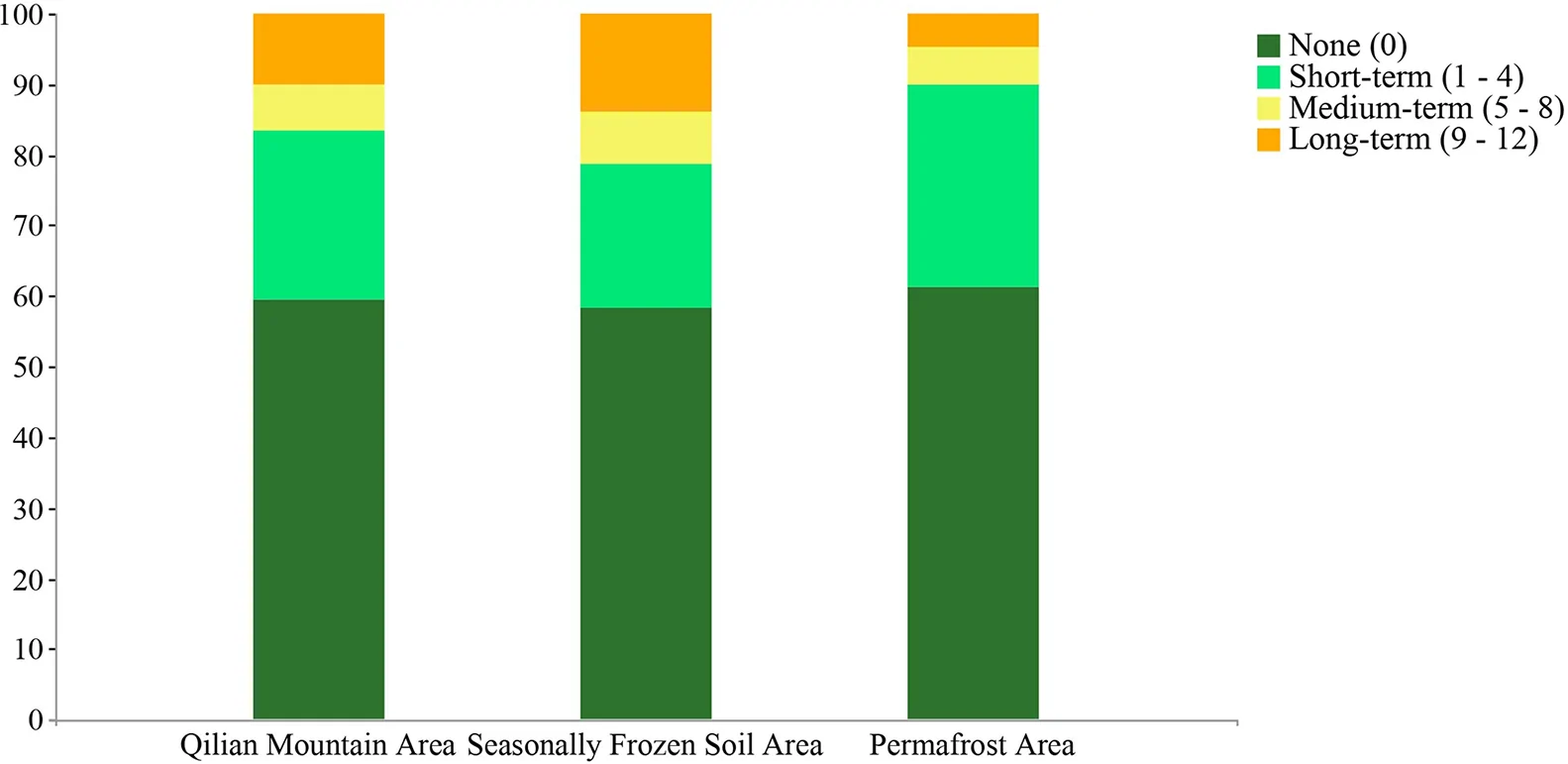
Cumulative-type percentage Bars show the percentage distribution of cumulative-response types within the Qilian Mountains, seasonally frozen soil area, and permafrost area. Percentages were calculated as the proportion of valid vegetation pixels assigned to each cumulative category within the corresponding region. Each bar sums to 100%.

**Figure 15:**
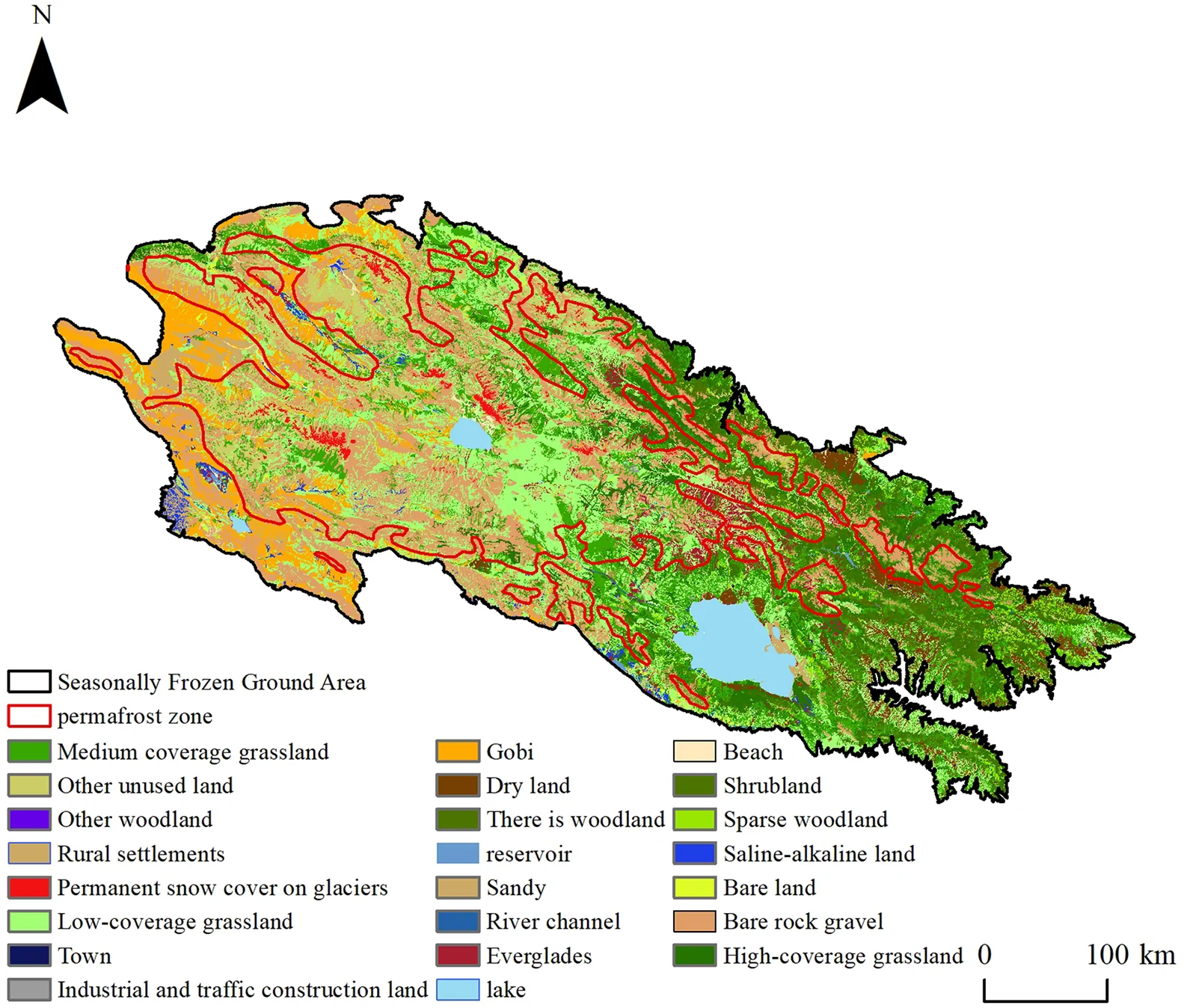
Distribution of land types in Qilian Mountains

**Figure 16:**
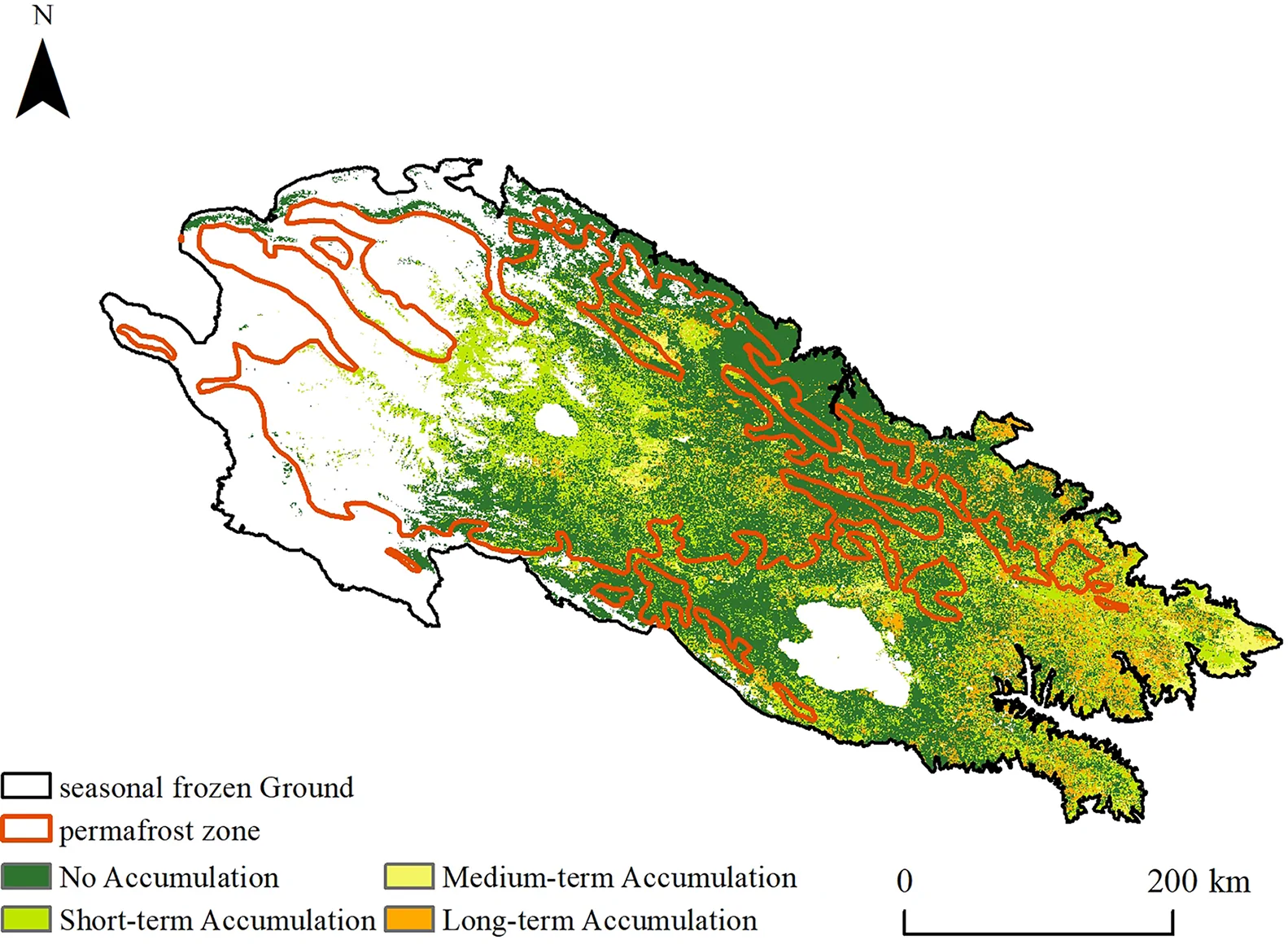
Cumulative type of Qilian Mountain Area

### Limitations of the study

The eddy-covariance flux data obtained in this study (Table 1) did not include directly observed evapotranspiration (ET); therefore, the FAO Penman-Monteith equation was used to calculate reference evapotranspiration^40^ (ET0) for site-level validation of MODIS-derived WUE. The strong Pearson’s correlation between site-level WUE (GPP/ET0) and MODIS-derived WUE (*r* > 0.895, *p* < 0.01) indicates that MODIS products can reliably capture the relative spatiotemporal patterns of WUE in the Qilian Mountains. The precondition for this statistical relationship to hold is that ET0 and actual ET are driven by common meteorological factors, and in the absence of extreme soil moisture deficit, their temporal variation directions at the growing-season scale tend to be consistent. Independent support for this inference is provided by measured studies of alpine meadows on the northeastern edge of the Qinghai-Tibetan Plateau: observations based on large weighing lysimeters show that actual ET is significantly correlated with ET0 calculated by the FAO-56 PM equation (*r* = 0.76, *p* < 0.01) under conditions where soil moisture is not limiting.^41^

**Table 1:** Flux sites within the HRB with time series data extending beyond one year^51^

ID	Name	Site_ID	Longitude (◦E)	Latitude (◦N)	Landcover	Dominant plant	Elevation (m)	Period	Stream
1	Jingyangling site	JYL	101.116	37.8384	Alpine grassland	Kobresia pygmaea	3750m	2018- Now	Up
2	Arou super site	ARS	100.4643	38.0473	Alpine grassland	Kobresia pygmaea	3033m	2013- Now	Up
3	Dadongshu site	DDS	100.2421	38.0142	Alpine grassland	Kobresia pygmaea	4148m	2015- Now	Up
4	Dashalong site	DSL	98.9406	38.8399	Alpine marshland	Kobresia pygmaea	3739m	2013- Now	Up
5	Arou site	ARo	100.4646	38.0443	Alpine grassland	Kobresia pygmaea	3033m	2008–2011	Up
6	Guantan site	GTa	100.2500	38.5333	Subalpine forest	Picea crassifolia	2835m	2010–2011	Up

It is worth noting that the correlation between WUE and WUE’ in this study (*r* > 0.895; Figure 17) is higher than the correlation between ET and ET0 (*r* = 0.76). This is a known statistical property of ratio variables: the correlation between two ratios sharing the same numerator (GPP) is structurally higher than the correlation between their denominators due to the variance contribution of the shared numerator. This effect precisely indicates that the relative spatiotemporal patterns of WUE in this study area are primarily driven by the interannual variability of GPP and are relatively insensitive to the choice of denominator (ET or ET0), so the marginal impact of substituting ET0 for ET on the validation of WUE relative patterns is limited. Climate records for the Qilian Mountains over the past 60 years show that regional consecutive dry days have decreased significantly, and precipitation in summer and autumn has increased,^42^^,^^43^ indicating that the overall moisture conditions in the study area are relatively stable; no persistent extreme drought events occurred during the validation period of this study, and the above preconditions were basically met during the study period.

**Figure 17:**
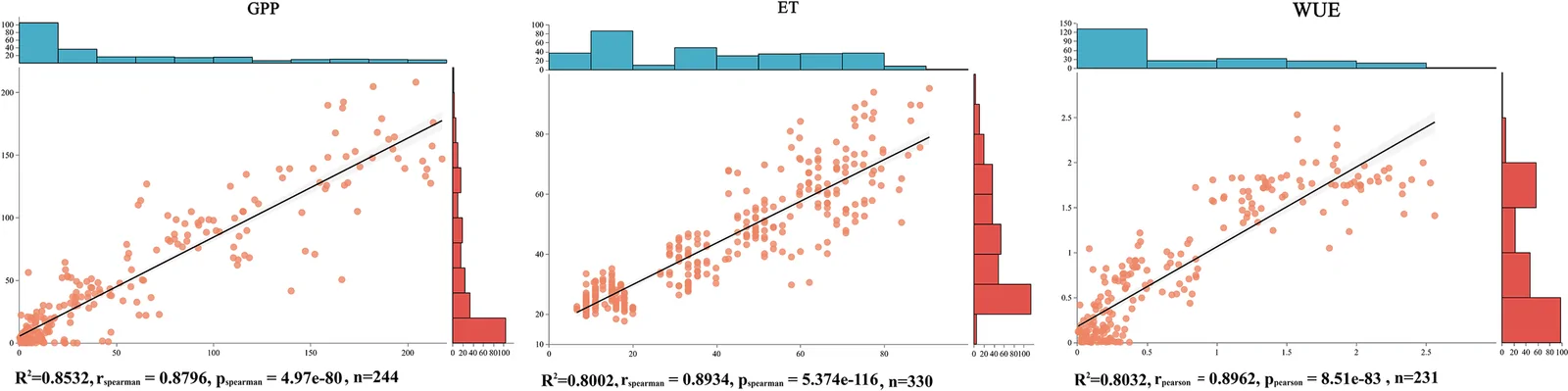
Validation of GPP, ET, and WUE data accuracy based on eddy covariance sites Each point represents one paired monthly observation between an eddy-covariance flux site and the corresponding MODIS pixel. Data from the six flux sites were pooled for the validation analysis. Pearson’s correlation coefficients are shown in each image.

However, it should be pointed out that ET0 and actual ET are not the same variable. ET0 represents atmospheric evaporative demand under standardized reference conditions,^40^ while actual ET is additionally constrained by soil moisture availability and canopy stomatal conductance.^44^ Under water-limited conditions, actual ET may fall below atmospheric evaporative demand.^45^ In the permafrost region of the Qilian Mountains, the thin active layer and limited meltwater supply constrain soil moisture availability, and this discrepancy may be most significant during dry periods, meaning that the reliability of the validation is relatively weaker under extreme drought conditions. It should be noted that the high correlation between MODIS ET and ground ET0 (*r* = 0.893) partly stems from the shared meteorological driving factors and cannot serve as independent evidence for substituting ET0 for ET. Nevertheless, the high correlation between MODIS ET and ET0 still provides basic consistency information on ET spatiotemporal patterns, and the independent validation results for all three components (GPP, ET, and WUE; *r* > 0.87) jointly indicate the overall reliability of MODIS products. The validation logic of this study is primarily built upon the physical mechanism that ET and ET0 are driven by common meteorological factors—that is, both share major driving factors such as net radiation, air temperature, and vapor pressure deficit, and exhibit temporal covariation at the growing-season scale—rather than relying on this statistical correlation. Based on this physical mechanism, the relative response patterns of WUE to drought identified in this study—that is, the trends of WUE change with intensifying drought—still have explanatory power in characterizing multi-year average and interannual variation relative patterns, although the precision of their absolute values is limited by the inherent uncertainty of ET0 substitution validation.

Therefore, in future research, it is necessary to further strengthen ground validation studies of remote sensing products in alpine cryosphere environments. Direct ET measurements obtained through independent methods, such as weighing lysimeters and sap flow sensors, will help quantify the magnitude of ET0-ET differences across different permafrost regimes and provide a more reliable basis for validating remote sensing evapotranspiration products in these data-scarce regions.

## Resource availability

### Lead contact

Requests for further information and resources should be directed to and will be fulfilled by the lead contact, Dawei Wang (giswang@163.com).

### Materials availability

This study did not generate new unique reagents.

### Data and code availability

• This paper analyzes existing, publicly available data. MODIS GPP (MOD17A2) and MODIS ET (MOD16A2) data are publicly available from NASA EOSDIS LP DAAC (https://lpdaac.usgs.gov/).
• The self-calibrated Palmer Drought Severity Index (scPDSI) dataset is publicly available from the Climate Research Unit, University of East Anglia (https://crudata.uea.ac.uk/cru/data/drought/).
• The post-processed carbon flux dataset for Heihe River Basin eddy covariance stations is publicly available from the National Tibetan Plateau Data Center at https://doi.org/10.11888/Terre.tpdc.301321.
• Permafrost distribution data for the Qilian Mountains are available from the National Tibetan Plateau Data Center (https://doi.org/10.11888/Geocry.tpdc.270456).
• Land use and land cover data are publicly available from the Resource and Environment Science and Data Center, Chinese Academy of Sciences (https://www.resdc.cn/).
• SRTM DEM data are publicly available from NASA Earthdata (https://earthdata.nasa.gov/).
• Meteorological station data are available from the China Meteorological Administration (http://data.cma.cn/).
• Any additional information required to reanalyze the data reported in this paper is available from the lead contact upon request.
• This paper does not report original code.

## Acknowledgments

This research was jointly supported by the Yunnan Provincial Basic Research (202301AT070084 and 202301AT070085) and the West Yunnan University of Applied Sciences Talent Introduction Research Initiation Project (2023RCKY0001 and 2022RCKY0003).

## Author contributions

Conceptualization, L.L. and D.W.; methodology, Y.G. and J.D.; formal analysis, Y.G.; investigation, Y.G., Y.Z., D.J., and Y.G.; resources, T.L. and J.L.; data curation, Y.G. and D.J.; writing – original draft, Y.G.; writing – review and editing, L.L., Y.Z., D.J., J.D., Y.G., T.L., J.L., and D.W.; visualization, Y.G., Y.Z., and Y.G.; supervision, D.W.; project administration, D.W.; funding acquisition, L.L. and D.W.

## Declaration of interests

The authors declare no competing interests.

## STAR★Methods

### Key resources table

**Table:** 

REAGENT or RESOURCE	SOURCE	IDENTIFIER
**Deposited data**		

Heihe River Basin carbon flux dataset	National Tibetan Plateau Data Center	https://doi.org/10.11888/Terre.tpdc.301321
Permafrost distribution data of the Qilian Mountains	National Tibetan Plateau Data Center	https://doi.org/10.11888/Geocry.tpdc.270456
MODIS GPP (MOD17A2)	NASA LP DAAC	https://earthdata.nasa.gov/
MODIS ET (MOD16A2)	NASA LP DAAC	https://earthdata.nasa.gov/
scPDSI (global gridded drought index)	Climatic Research Unit, University of East Anglia	https://crudata.uea.ac.uk/
DEM (SRTM)	NASA	https://earthdata.nasa.gov/
Meteorological station data	China Meteorological Administration	http://data.cma.cn/
Land use and land cover data	Resource and Environment Science and Data Center, Chinese Academy of Sciences	https://www.resdc.cn/
Data Resource	This study	Data S1

**Software and algorithms**		

MODIS Reprojection Tool (MRT)	NASA	https://lpdaac.usgs.gov/tools/modis-reprojection-tool/
Anusplin 4.2	Australian National University	https://fennerschool.anu.edu.au/research/products/anusplin
EToCalculator (FAO Penman-Monteith)	FAO	https://www.fao.org/land-water/water/water-management/en/
ArcGIS 10.x	ESRI	https://www.esri.com/

### Method details

#### Study area

The Qilian Mountains region is located at the junction of the Tibetan Plateau, the Loess Plateau, and the Mongolian Plateau (93°30′–103°54′E, 35°51′–39°58′N), with a mountainous area of 16.90 × 10^4^ km^2^ and an elevation ranging from 2004 to 5716 m (Figure 1). The terrain is higher in the west and lower in the east, with a northwest-southeast orientation of the mountain ranges. The climate of the Qilian Mountains region is characterized as a typical continental cold semi-humid mountain climate, with cold and dry winters, and cool and moist summers. The annual precipitation is maintained at around 400 mm, with the majority of precipitation occurring from May to September,^35^ decreasing from 600 mm in the east to less than 100 mm in the west. The vegetation types in the permafrost areas of the Qilian Mountains are mainly grasslands and deserts, while the seasonally frozen soil areas are characterized by evergreen coniferous forests, mixed forests, savannas, grasslands, and croplands. The high elevation of the Qilian Mountains region results in a cold climate. As the main distribution area of high-mountain permafrost in Northwest China, the hydrological system is primarily replenished by glacier meltwater, and the permafrost area accounts for 47.51% of the total area of the Qilian Mountains.^23^

#### Remote sensing data

GPP and ET data were obtained from the MODIS products (MOD17A2, MOD16A2) released by the National Aeronautics and Space Administration (NASA) (https://www.earthdata.nasa.gov/). The data have a spatial resolution of 500 m and a temporal resolution of 8 days, covering the period from 2001 to 2020. The GPP data algorithm is based on radiation use efficiency, with detailed calculations described in (Running S. W., 2004).^46^ The ET data algorithm is based on the Penman-Monteith equation, which comprehensively considers soil surface evaporation, interception evaporation of precipitation by the canopy, and plant transpiration. Detailed information can be found in (Mu et al., 2011).^47^ The MODIS data were batch-mosaicked and reprojected using the MRT (MODIS Reprojection Tool) software. Monthly composites of the reprojected data were created and clipped to the Qilian Mountains region. The accuracy of MODIS GPP and ET products has been validated through flux tower sites in multiple regions worldwide and has been confirmed in numerous studies.^48^^,^^49^^,^^50^

#### Remote sensing data validation

To validate the accuracy of MODIS GPP/ET global data in the study area, the post-processing carbon flux dataset from the Heihe River Basin eddy flux stations^51^ was used. The validation data consisted of monthly measured total primary productivity (GPP) from six eddy flux stations located in the Qilian Mountains. Detailed station information is provided in the Table 1.

To estimate the reference evapotranspiration (ET_0_) at six eddy covariance flux stations in the Qilian Mountains, this study first extracted measured wind speed, average temperature, and relative humidity data from each station. Surface meteorological observations from Gansu and Qinghai provinces were then spatially interpolated using Anusplin 4.2, with elevation as a covariate, to generate monthly 500 m resolution raster datasets of maximum temperature, minimum temperature, and sunshine duration that match the spatial resolution and projection of MODIS products. Pixel values corresponding to each flux station location were extracted accordingly. Finally, ET_0_ was computed using the EToCalculator software, and water use efficiency (WUE) was derived as the ratio of gross primary production (GPP) to ET_0_ (WUE = GPP/ET_0_) based on GPP data obtained from the flux stations.

The ground-observed GPP, ET, and WUE data from six eddy covariance flux stations were respectively compared and validated against the corresponding MODIS product data at their spatial locations (Figure 17). Results showed that the correlation coefficients between the station-based measurements and MODIS data at corresponding pixels all exceeded 0.8. Among them, WUE exhibited the highest correlation (Pearson r > 0.895, *p* < 0.01), followed by ET (r = 0.893, *p* < 0.01), while GPP also reached above 0.87 (*p* < 0.01). In terms of regression evaluation metrics, the mean squared errors (MSE) for GPP, ET, and WUE were 637.72, 142.83, and 0.12, respectively; the root mean squared errors (RMSE) were 25.25, 11.95, and 0.34; and the mean absolute errors (MAE) were 16.04, 9.91, and 0.26. Overall, the MODIS-estimated GPP and ET align with the actual ecohydrological characteristics of the Shiyang River Basin, indicating that MODIS data have high accuracy in this region and can reasonably reflect the actual conditions of the Qilian Mountain ecosystem.

#### scPDSI data

The scPDSI data are sourced from the global gridded drought index dataset provided by the Climate Research Unit of the University of East Anglia (https://crudata.uea.ac.uk/). The dataset has a spatial resolution of 0.5 ° × 0.5 ° and a temporal resolution of monthly, covering the period from 2001 to 2020. For detailed information on the scPDSI algorithm, please refer to the literature.^52^ To ensure consistency in spatial resolution, the Anusplin4.2 software was utilized, incorporating elevation information as a covariate, to interpolate the data using a variable interpolation method. This process yielded monthly scPDSI data for the Qilian Mountains with the same pixel size and projection as the MODIS data. Given that the scPDSI dataset has a spatial resolution of 0.5 ° × 0.5 °, it was spatially interpolated to a 500 m resolution using the Anusplin software to match the spatial resolution of the MODIS GPP/ET data.To assess the accuracy of the spatial interpolation results, 20% of the grid cells within the study area were randomly selected as validation data before interpolation. A comparison between the interpolated values and the original values (Figure 7) revealed a correlation coefficient (R) of 0.93. The overall standard deviation of the interpolated scPDSI results (2.09) was slightly higher than that of the original values, with a root-mean-square error (RMSE) of 0.75. Overall, the scPDSI data interpolated using Anusplin4.2 demonstrated high simulation accuracy and are consistent with the actual conditions of the Qilian Mountains region.

### Quantification and statistical analysis

#### Calculation of WUE

In this study, water use efficiency was calculated using the commonly used method in ecosystem studies, which is the ratio of total ecosystem gross primary productivity to total evapotranspiration^53^:

(Equation 1) $$\text{WUE}=\frac{\text{GPP}}{ET}$$

Where WUE is the water use efficiency (gC·m^−2^·mm^−1^); GPP is the total ecosystem gross primary productivity (gC·m^−2^); ET is the total ecosystem evapotranspiration (mm).

#### Trend analysis

Using univariate linear regression analysis at each pixel, we simulated the monthly WUE from 2001 to 2020 to obtain the trend, with the calculation formula as follows^54^:

(Equation 2) $$\theta_{Slope}=\frac{n\times \left(\sum_{i=1}^{n}i\times C_{i}\right)-\left(\sum_{i=1}^{n}i\right)\left(\sum_{i=1}^{n}C_{i}\right)}{n\times \sum_{i=1}^{n}i^{2}-{\left(\sum_{i=1}^{n}i\right)}^{2}}$$

Where n is the number of years in the time period (*n* = 20); *θ*_slope_ is the slope of the trend; *C*_*i*_ is the WUE for year *i* A negative *θ*_slope_ indicates a decreasing trend in WUE, while a positive *θ*_slope_ indicates an increasing trend in WUE.

#### Correlation analysis between WUE and scPDSI

Pearson correlation analysis was used to calculate the correlation coefficient between WUE and scPDSI on a yearly basis, with the calculation formula as follows^54^:

(Equation 3) $$R_{xy}=\frac{\sum_{i=1}^{n}\left[\left(x_{i}-\bar{x}\right)\left(y_{i}-\bar{y}\right)\right]}{\sqrt{\sum_{i=1}^{n}{\left(x_{i}-\bar{x}\right)}^{2}\sum_{i=1}^{n}{\left(y_{i}-\bar{y}\right)}^{2}}}$$

Where *R*_*xy*_ is the correlation coefficient between the two variables; *x*_*i*_ is the WUE for each month of the i-th year; *y*_*i*_ is the scPDSI for each month of the i-th year; $\bar{x}$ is the average of WUE for each month and year; $\bar{y}$ is the average of scPDSI for each month and year; *i* is the sample size. A positive value of *R*_*xy*_ indicates a positive correlation between WUE and scPDSI, while a negative value indicates a negative correlation.

#### Significance test

The significance of the trends in WUE and the correlation coefficients with scPDSI was tested using the F-test. The significance represents the reliability of the trends and correlation coefficients, with the calculation formula as follows^54^:

(Equation 4) $$F=U\times \frac{n-2}{Q}$$

Where $U={\sum_{i=1}^{n}\left({\hat{y}}_{i}-\bar{y}\right)}^{2}$ is the regression sum of squares, $Q={\sum_{i=1}^{n}\left(y_{i}-{\hat{y}}_{i}\right)}^{2}$ is the residual sum of squares, *y*_*i*_ is the WUE value for month i, $\hat{y_{i}}$ is the fitted value, *y*_*i*_ is the average WUE value over multiple years for each month, and *n* = 20 is the number of years. For the temporal and spatial variation trends of WUE, the following six change levels were determined based on the F-test results: extremely significant decrease(*θ*_slope_ < 0, *p* < 0.01), significant decrease (*θ*_slope_ < 0, *p* < 0.05), non-significant decrease (*θ*_slope_ < 0, *p* > 0.05); non-significant increase (*θ*_slope_ > 0, *p* > 0.05), significant increase (*θ*_slope_ > 0, *p* < 0.05), extremely significant increase (*θ*_slope_ > 0, *p* < 0.01). For the correlation between WUE and scPDSI, the following six change levels were classified based on the F-test results: extremely significant negative correlation (R < 0, *p* < 0.01), significant negative correlation (R < 0, *p* < 0.05), non-significant negative correlation (R < 0, *p* > 0.05); non-significant positive correlation (R > 0, *p* > 0.05), significant positive correlation (R > 0, *p* < 0.05), extremely significant positive correlation (R > 0, *p* < 0.01).

#### lag effect of scPDSI on WUE

The Pearson correlation coefficient (R) is used to study the magnitude and temporal scale of the lagged effect of drought on grassland GPP.^55^ It ranges from −1 to 1, representing negative to positive correlations. To ensure the comparability of the correlation results, this study selected monthly WUE and 1-month scPDSI data for the Qilian Mountains from 2001 to 2020. For each month, WUE was combined with the scPDSI from i months prior (0 ≤ i ≤ 12), forming a series. Then, the correlation coefficient R was calculated for each lag timescale i, yielding 12 correlation coefficients for each pixel (Equation 5). For example, a 3-month lag was analyzed by correlating monthly WUE data from January to July (2001–2020) with scPDSI data from April to October (2001–2020), and this was extended up to a 12-month lag. Finally, the maximum correlation coefficient Rmax_lag was selected, and the corresponding lag month was considered the magnitude and temporal scale of the lagged effect for that pixel (Equation 6). If the Rmax_lag between monthly WUE and 1-month scPDSI occurs in April, then the lagged response timescale of vegetation WUE to scPDSI is recorded as 4 months, indicating that drought conditions 4 months prior have a significant impact on the changes in vegetation WUE.

(Equation 5) $$R_{i}=corr\left(WUE,iscPDSI\right)0\leq i\leq 12$$

(Equation 6) $$R_{\max\_lag}=\max\left(R_{i}\right)\;0\leq i\leq 12$$

where WUE represents the monthly time series of WUE with a lag of i months from 2001 to 2020, scPDSI is the 1-month scPDSI time series lagged by i months, and R is the Pearson correlation coefficient for a lag of i months. Based on the results, the lagged effects were classified into the following four types: no lagged effect (lag month = 0), short-term lagged effect (1 ≤ lag month ≤4), medium-term lagged effect (5 ≤ lag month ≤8), and long-term lagged effect (9 ≤ lag month ≤12).

#### Cumulative effect of scPDSI on WUE

To assess the cumulative impact of drought on grassland WUE, the Pearson correlation coefficient between monthly WUE and cumulative scPDSI was used to obtain the scPDSI timescale corresponding to the maximum correlation.^55^ Unlike the lagged effect, which uses only the lagged scPDSI, the cumulative effect requires scPDSI data from 0 to 12 months. Therefore, the 1- to 12-month scPDSI dataset and WUE pixel values from 2001 to 2020 were used to determine the correlation.

First, the WUE time series were correlated with the scPDSI time series at an m-month scale (0 ≤ m ≤ 12) to calculate R (Equation 7). Then, the cumulative months of scPDSI with the maximum correlation R_max__acc to WUE were considered the timescale of the cumulative effect (Equation 8), and R_max__acc was determined as the magnitude of the cumulative effect. For example, if the correlation between monthly WUE and 3-month scPDSI is the highest, the timescale of the cumulative effect is recorded as 3 months, indicating that the accumulated drought conditions over the past 3 months have the greatest impact on vegetation WUE.

(Equation 7) $$R_{m}=corr\left(WUE,mscPDSI\right)0\leq M\leq 12$$

(Equation 8) $$R_{\max\_acc}=\max\left(R_{m}\right)0<m\leq 12$$

Based on the results, the cumulative effect is classified into four types: no cumulative effect (cumulative month = 0), short-term cumulative effect (1 ≤ cumulative month ≤4), medium-term cumulative effect (5 ≤ cumulative month ≤8), and long-term cumulative effect (9 ≤ cumulative month ≤12).

## Supplemental information

Data S1. Including monthly spatial patterns of water use efficiency (WUE), gross primary productivity (GPP), and evapotranspiration (ET) across the Qilian Mountains from 2001 to 2020, drought index (scPDSI), and statistical results of lagged and cumulative effects
